# Cross-population enhancement of PrediXcan predictions with a gnomAD-based east Asian reference framework

**DOI:** 10.1093/bib/bbae549

**Published:** 2024-10-23

**Authors:** Han-Ching Chan, Amrita Chattopadhyay, Tzu-Pin Lu

**Affiliations:** Institute of Epidemiology and Preventive Medicine, Department of Public Health, National Taiwan University, Room 518, No. 17, Xu-Zhou Road, Taipei 10055, Taiwan; Institute of Epidemiology and Preventive Medicine, Department of Public Health, National Taiwan University, Room 518, No. 17, Xu-Zhou Road, Taipei 10055, Taiwan; Institute of Epidemiology and Preventive Medicine, Department of Public Health, National Taiwan University, Room 518, No. 17, Xu-Zhou Road, Taipei 10055, Taiwan; Institute of Health Data Analytics and Statistics, Department of Public Health, National Taiwan University, Room 518, No. 17, Xu-Zhou Road, Taipei 10055, Taiwan

**Keywords:** transcriptome-wide association studies (TWAS), population diversity, PrediXcan, single-nucleotide polymorphism

## Abstract

Over the past decade, genome-wide association studies have identified thousands of variants significantly associated with complex traits. For each locus, gene expression levels are needed to further explore its biological functions. To address this, the PrediXcan algorithm leverages large-scale reference data to impute the gene expression level from single nucleotide polymorphisms, and thus the gene-trait associations can be tested to identify the candidate causal genes. However, a challenge arises due to the fact that most reference data are from subjects of European ancestry, and the accuracy and robustness of predicted gene expression in subjects of East Asian (EAS) ancestry remains unclear. Here, we first simulated a variety of scenarios to explore the impact of the level of population diversity on gene expression. Population differentiated variants were estimated by using the allele frequency information from The Genome Aggregation Database. We found that the weights of a variants was the main factor that affected the gene expression predictions, and that ~70% of variants were significantly population differentiated based on proportion tests. To provide insights into this population effect on gene expression levels, we utilized the allele frequency information to develop a gene expression reference panel, ***Predict Asian-Population*** (PredictAP), for EAS ancestry. PredictAP can be viewed as an auxiliary tool for PrediXcan when using genotype data from EAS subjects.

## Introduction

Genome-wide association studies (GWASs) are designed to identify genetic variants associated with phenotypes by testing the differences in the allele frequency across thousands of genetic variants [1]. Variants with a strong association enables a better understanding of the complex biology underlying traits and diseases. Over the past decade, many GWASs have successfully identified variants associated with complex traits. However, the interpretation of the biological mechanisms derived from these discoveries remains unclear. First, most of the discovered variants are located in non-coding regions of the genome, suggesting an indirect regulatory relationship in cellular functions [2, 3]. Second, the identification of causal variants is hindered by linkage disequilibrium, which causes non-causal variants to be significantly associated with the phenotype. It is thus challenging to decipher the mechanistic underpinnings of GWAS results.

Expression quantitative trait loci (eQTL) analysis is an effective way to leverage transcriptomic data to prioritize causal variants that are truly associated with a complex trait [4, 5]. Integrating gene expression level information with GWAS results, allows a better understanding of genetic regulatory mechanisms. Transcriptome-wide association studies (TWASs) enrich the eQTL framework by utilizing a reference panel to evaluate the genetic regulatory relationship. Prediction models can then be developed to impute the gene expression utilizing significantly associated single nucleotide polymorphisms (SNPs) encompassing a tissue, and subsequently, the gene-trait association can be tested to identify the candidate causal genes. Several studies have successfully identified novel risk-associated genes in complex diseases using TWAS results [6–9]. However, these studies have also drawn attention to the need for diversity in populations. Increasing evidence suggests that gene expression patterns differ among and between human populations belonging to different ethnicities [10]. However, the impact of this variation on human diseases has not been thoroughly investigated. This is partly due to the absence of a standardized protocol for estimating biogeographical ancestry from gene expression studies. As of 2023, according to the GWAS catalog, ~86.6% of participants are of European ancestry in GWAS studies [11]. The lack of consideration of ethnic diversity in genomic studies makes it difficult to gain mechanistic insights into targeted genes, further reducing their applicability when utilized in other populations. This disparity is an inevitable outcome of the Eurocentric biases present in GWASs. For example, the polygenic risk score developed using the European population achieves a 2-fold accuracy compared to when applied on the subjects from East Asian (EAS) ancestry [12]. To expand the generalizability of biomarkers for all populations, it is necessary to consider population differences in biological discovery.

The PrediXcan method is one of the several well-known TWAS methods, where an elastic net regression model is trained to obtain weights for each cis-variant utilizing a reference data consisting of genotype and transcriptome information [13]. The resulting model can predict gene expression from individual-level genotype data within a specific tissue. The advantages of PrediXcan include smaller multiple testing burden compared to GWASs, higher interpretability of individual-level gene expression, aggregation of the small effects of individual genetic variants, and the ability to generate tissue-specific models that incorporate the patterns of regulation in different tissue types. However, despite the existence of a large-scale reference data for training, the performance of PrediXcan may not be consistent across diverse populations with distinct genetic backgrounds. The majority of subjects in the Genotype-Tissue Expression (GTEx) Project analyzed by PrediXcan, were of European ancestry. Different allele frequency patterns in different populations may affect the prediction performance; a recent work demonstrated that the similarity of ancestry plays a critical role in the accuracy of prediction and the power of association detection [14, 15].

To address this issue, in this study we first explored and confirmed the impact of population diversity on gene expression prediction. We leveraged the weights across all tissue types from PrediXcan to simulate a variety of scenarios, utilizing different parameters such as the number of variants, the minor allele frequency (MAF), the proportion of population-differentiated variants and weight groups. We also tested whether the genetic variants have significant differences in allele frequency between European and EAS ancestry. Secondly, to take into consideration the effect, that population differences may have on PrediXcan prediction, we developed a gene expression reference panel, specifically for the EAS ancestry, by utilizing allele frequency information derived from The Genome Aggregation Database (gnomAD) in order to capture the systematic specificities of the population. Finally, we built an R package named ***Predict Asian-Population*** (PredictAP) for users to conduct evaluation and comparison analyses using prediction results from PrediXcan.

## Methods

### Datasets

**Figure 1:**
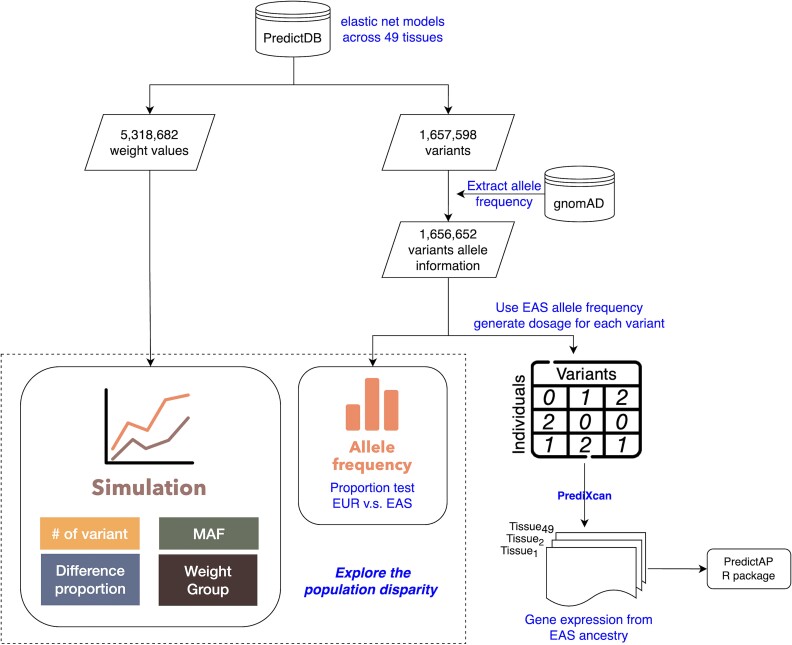
The flow chart of the simulation and analysis. A total of 49 tissues from the PredictDB database (https://predictdb.org/) were acquired. Allele frequency information from gnomAD v2.1.1 for DNA variants were retrieved. Simulation box denotes that simulation analyses were conducted utilizing parameters, number (#) of variants, MAF, proportion of population-differentiated variants (difference proportion) and weight group. Allele frequency box denotes allele frequencies of population differentiated variants (determined by proportion test). Allele frequency information of population differentiated variants that were specific to EAS were utilized to calculate dosages, which were fed into PrediXcan to predict gene expression 500 times for each tissue specific variant, to construct empirical distribution(s). These distributions are used to provide summary parameters, that constitute PredictAP (R-package).

An overview of the workflow implemented in this study is demonstrated via Fig. 1. It gives a comprehensive view of the process that was utilized in this study to evaluate the effect that population diversity may potentially have on gene expression prediction using PrediXcan and consequently the workflow to establish gene expression reference levels for EAS ancestry. To that end, trained gene expression models for 49 tissues from the PredictDB database (https://predictdb.org/) [13, 16, 17] and allele frequency information from gnomAD v2.1.1 for DNA variants from the EAS and European ancestry groups [18], were retrieved, respectively.

Additionally two real datasets were downloaded from Gene Expression Omnibus (GSE33356 and GSE26853) in order to demonstrate how users can utilize PredictAP to evaluate the accuracy of gene expression predictions using PrediXcan. GSE33356 contains paired SNP array data and gene expression data from 61 non-smoking lung cancer patients in Taiwan and GSE26853 contains SNP array data from blood DNAs of 95 patients with gastric cancer.

### Simulation studies to identify parameters that represent population diversity

Extensive simulation experiments were performed to explore the differences, if any, of the impact that SNPs from the EAS and European populations have on gene expression prediction. Towards that end, different scenarios were considered. In the PrediXcan algorithm, the gene expression is determined by the summation of variants allele numbers multiplied by their corresponding weights (**E1**)


(E1) \begin{equation*} {Y}_s=\sum_k{w}_{k,s}{X}_k \end{equation*}

where, ${w}_{k,s}$ is the weight of variant k for gene s, ${X}_k$ is the number of minor alleles of variant k.

Therefore, based on E1, the parameters, *number of variants*, *MAF*, *proportion of population-differentiated variants*, and weights, were selected for simulations. The number of variants used for predicting the gene expression, along with the MAFs were used to simulate the minor allele for each variant. MAF values were chosen at intervals of 0.1 to represent a range of allele frequencies, from common to rare variants, which helps in understanding how the frequency of minor alleles affects gene expression prediction. This was done to ensure that both low-frequency and high-frequency alleles are adequately represented. Besides, the increment setting of 0.1 allowed us to observe how gradual changes in allele frequencies affected the prediction, providing clearer insights into the relationship between MAF and gene expression prediction. Lastly, considering the computational practicality, 0.1 was a strategic choice that balanced thoroughness and efficiency. On the other hand, by controlling and varying the levels of the population-differentiated variants, the impact of population differences on gene expression prediction was evaluated. For instance, understanding scenarios like, the extent of impact on the gene expression prediction when the proportion of population-differentiated variants were high along with high corresponding weights, would help decode how genetic diversity and the distribution of allele frequencies influence the performance of predictive models.

**Figure 2:**
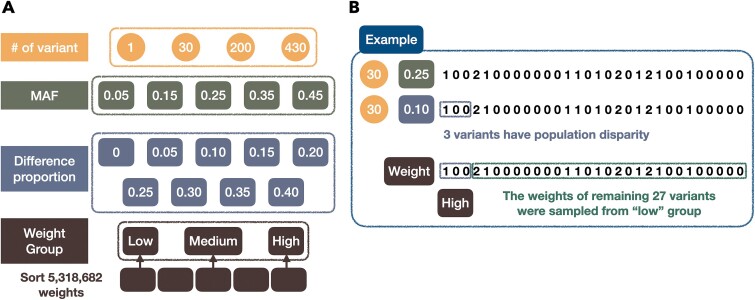
The schematic diagram describing the parameters involved in data simulations.

Figure 2 is a schematic representation along with explanations of the process of generating simulated data that involves four parameters. The first parameter is the number of variants (Fig. 2A: first panel). The estimated number of variants from the gene expression models across all tissues in PrediXcan, which was utilized to predict a gene, ranged from a minimum of 1 to ~200, with an average of 30, while the maximum number used across all tissues is ~430. Thus, we conducted simulations with 1, 30, 200, and 430 variants. Next, MAF (Fig. 2A, second panel from the top) ranging from 0.05 to 0.45 with increments of 0.1, resulting in five possible values were utilized. By varying the number of variants and MAF, we can generate a set of variant data. For example, in Fig. 2B top panel, we specified the gene expression prediction to be based on 30 variants, with each variant having a MAF of 0.25, allowing us to simulate the number of minor alleles for each variant.

The third parameter ‘difference proportion’, represents the proportion of variants that exhibit population differences (Fig. 2A, third panel). This parameter was ranged from 0 to 0.4, with increments of 0.05, resulting in nine possibilities. For instance, in Fig. 2B second panel, a difference proportion of 0.1 implies that out of the 30 variants, three exhibit population differences (highlighted). The final parameter aims to determine the weights of these differing variants from high, medium, or low groups (Fig. 2A, bottom panel). First, the absolute pooled weights across tissues were calculated, where the absolute function avoided offset during the linear combination of variant dosages. The weights across all the 49 tissues were sorted and merged into a vector, and partitioned into five equal segments. The first, third, and fifth segments represented the low, medium, and high weight levels (Fig. 2A). These weight levels were applied to all population-differentiated variants, aiming to investigate the impact of gene expression. For instance, in Fig. 2B, if the weight group is ‘high’, it implies that the weights of the three differing variants are randomly selected from the high group. As for the remaining 27 variants, their weights are fixed and randomly selected from the ‘low’ group.

Overall, our simulation datasets were constructed utilizing combinations of the abovementioned four parameters, where each combination was repeated 500 times. All the simulations were conducted using R4.3.2. Libraries, data.table, tidyr, ggplot2, and ggpubr from R were utilized, where data.table and tidyr were utilized to import and calculate weights, respectively, and both ggplot2 and ggpubr to make figures. The simulation results were judged across MAFs and the 500 permutations were utilized to obtain the empirical cumulative distribution function (ECDF).

### Proportion test on the population differentiated variants

In addition to comparing the tissue-specific gene expression, we sought to determine the number of variants with significant differences in allele frequency between EAS and European populations. We extracted the allele frequency information from gnomAD, and only those variants with allele information in both populations, were retained for analysis (Fig. 1). For each variant, the proportion test was applied with a p-value threshold set as 5$\times$10^−8^ to adjust for multiple testing. Variants that passed the test and had an allele frequency difference, between the two populations, to be greater than 0.05, were defined as significant population-differentiated variants.

### Developing gene expression reference for east Asian ancestry

A vital issue in PrediXcan is that the gene expression model was trained using the GTEx project, which comprises subjects of European ancestry. This creates a potential limitation when using PrediXcan to predict gene expression for the EAS population. Taking this into consideration, in this study a reference panel is established to enable evaluation of gene expression prediction by PrediXcan, specifically for the EAS population. Initially, we applied the MAF information from gnomAD to generate the dosage for each variant across 49 tissues (Fig. 1). This procedure was repeated 500 times to generate a distribution. Additionally, to ensure the stability of the gene expression reference, we compared results that were obtained via 500, 1000, and 2000 repetitive analyses. Subsequently, PrediXcan was performed to predict the gene expression utilizing EAS SNPs for each tissue. We also calculated the average and the dispersion (standard deviation) of the gene expression, along with the number of variants from each gene. Next an R package, PredictAP, was built under R4.3.2, that provides users with all of the above information thus enabling them to evaluate PrediXcan’s performance using the summary parameters from an empirical distribution and percentile rank of the gene expression, via the following steps.

1. Obtain predicted gene expression by utilizing Predixcan (GTEx Reference) for EAS population specific studies.
2. Feed in the PrediXcan output as an input file into ‘PredictAP’ R package as developed in this study.
3. Download the results to judge and evaluate the prediction as obtained from PrediXcan

**Figure 3:**
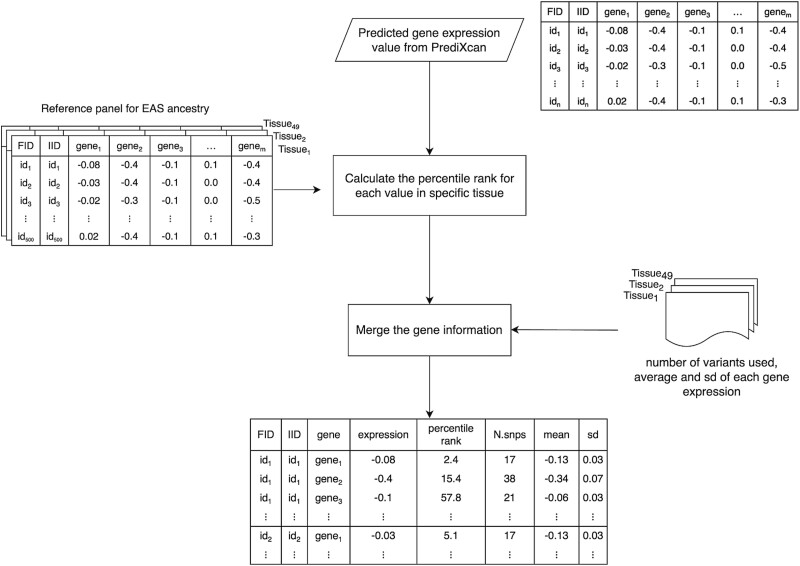
The algorithm of R package PredictAP. The input data is the predicted gene expression result from PrediXcan, consisting of family ID, individual ID, and the gene list corresponding to the tissue. For a given tissue, we calculate the percentile rank of each predicted gene expression compared to 500 gene expression reference values of EAS ancestry. Subsequently, we merge the gene information from the reference, including the number of variants used and average and standard deviation of gene expression. The output data is presented by subject to provide insight into population differences.

The algorithm of the package is illustrated in Fig. 3 and the package is available on the following link: https://www.space.ntu.edu.tw/navigate/a/#/s/53A4746DCB2C4B1E9E7E37AFEB17892A6BL.

### Evaluation of Predixcan results using PredictAP

Kolmogorov–Smirnov tests were employed to compare EAS and European (NFE) populations for each gene in each tissue. For calculations, the EAS values directly utilized the reference panel (500 values) while for the European population, we generated 500 PrediXcan predicted values using allele frequencies from the ‘European (non-Finnish)’ population in gnomAD. For multiple testing correction, the p-value threshold used in the package's result tables was set at 5×10^-8, which is used to decide the significance of the differences between the gene expression between the two populations and demonstrated in the R-package results. Additionally, a second evaluation was also done utilizing a Bonferroni corrected threshold but is not included in the R package.

### Application using external dataset

First, the lung cancer dataset with paired SNP array data and gene expression data was utilized for analyses. Inverse normalization was conducted on the gene expression data to mimic the normalization process that was done on GTEx data while training Predixcan to obtain normalized gene expression, GE_Real_(normalized). Next, genotype imputation utilizing Michigan Imputation Server and 1000 Genomes EAS panel as the reference were conducted on the raw SNP genotype data. The imputed SNPs were then fed into PrediXcan to predict lung tissue specific gene expression (GE_PrediXcan_). A correlation analysis between GE_Real_(normalized) and GEPrediXcan, was conducted to check the concordance between them to check the plausibility of external validation. Finally, GE_PrediXcan_ was evaluated against the confidence intervals (CI) provided by PredictAP, as reference.

## Results

### Overview of PrediXcan and gnomAD

Table S1 provides a summary of the number of genes with expression predicted by GTEx v8 elastic net models for each tissue. The minimum and maximum number of predicted genes were 1642 and 10 012 for the kidney cortex and tibial nerve, respectively. The number of variants used in these predictions were 60 744 and 259 465, respectively. The total number of unique genes and variants after integrating all 49 tissues were 1 657 598, out of which a total of 1 656 652 variants were with allele frequency information from gnomAD. The weight thresholds of the segments, for low, medium, and high levels were set as <0.0023, >0.0063 to <0.0135, and > 0.0298, respectively.

### Population diversity: Effect on gene expression prediction

**Figure 4:**
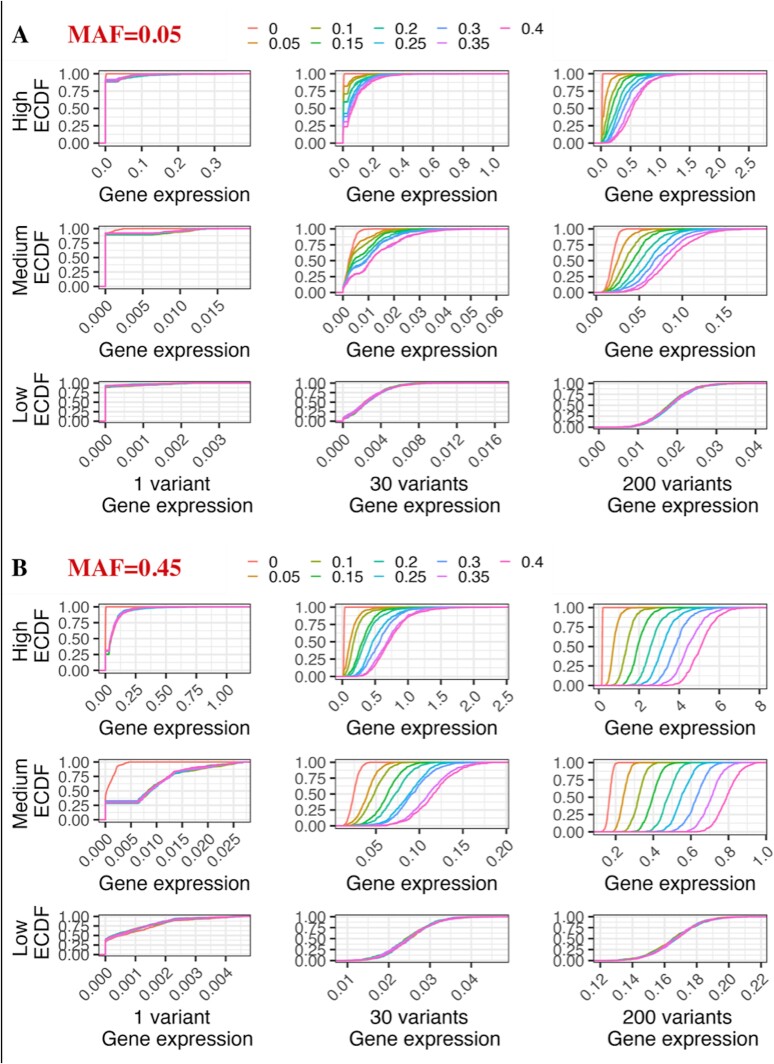
The ECDF curve of simulation results for (A) MAF equal to 0.05 and (B) MAF equal to 0.45. Given a fixed MAF, a total of nine combinations based on the number of variants used in a gene prediction (outer X-axis) and weight level of population-differentiated variants (outer Y-axis) are simulated. Each line shows the ECDF curve of predicted gene expression. The colors listed in the legend explain the proportion of population-differentiated variants. The closer lines mean the distribution of predicted gene expression was more similar.

Figures 4A and B and Figs. S1–S3 demonstrates the ECDF curves from the simulation results for MAFs, 0.05 and 0.45, respectively. It can be observed that with the increase in the proportion of population-differentiated variants, the space between the ECDF curves for different proportions, increased, implying that higher proportion of variants with population differences leads to higher gene expression. This is because, with an increasing number of variants, the differences due to population effects become more pronounced. The simulation result for 430 variants showed a similar trend (Fig. S4). However, the weight of the population-differentiated variants had the greatest impact on predicted gene expression. Table S2 shows the average difference and ratio of gene expression with zero percent as the baseline. Due to the broader range of gene expression at MAF = 0.45, the mean difference with 200 variants peaked at 4.95 at the high weight level. For a total of 30 and 430 variants, the mean difference peaked at 0.74 and 11.5, respectively. Moreover, at all of these differences, the gene expression at the high weight level with 30, 200, and 430 variants was ~ 30 times larger than the baseline, indicating the maximum impact of weights on PrediXcan prediction.

The proportion test was applied to compare the difference in allele frequency for each variant between EAS and European ancestry. Out of 1 656 652 variants, 48 were excluded because the allele frequency information was missing in one of the two populations. As a result, 1 158 742 (70%) significant variants were identified whose difference was >0.05.

### Gene expression reference for east Asian ancestry

To test the stability of gene expression reference panel, Kolmogorov–Smirnov tests were conducted to test whether the distribution of gene expression was significantly different for each gene for different repetitions (500, 1000, and 2000 times). After Bonferroni adjustment (for example, the significance level was set at *P* = 6.89E-06 by dividing 0.05 by 7252 where 0.05 is the nominal p-value and 7252 were the total genes for whole blood tissue), none of the distributions derived from the 500 simulations for each gene were significantly different from those derived from 1000 or 2000 simulations. Based on this finding, we utilized the results from 500 repetitions to represent the gene expressions in the EAS reference.

### PredictAP: The R package

**Figure 5:**
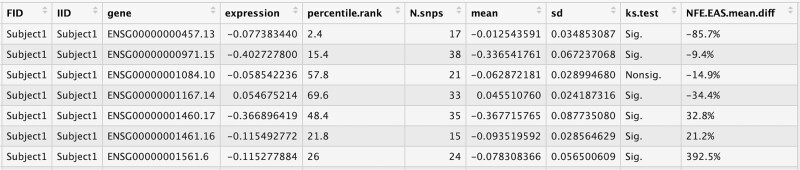
Screen shot of the output of R package PredictAP. The first four columns represent the original data as uploaded in the required format by the user. Columns 5–10 demonstrate the results as provided by PredictAP for the users to evaluate the prediction of gene expression by PredictAP.

In our R package algorithm, the gene expression values (from PrediXcan) as uploaded by users are compared to the 500 reference values, and a percentile rank is calculated which serves as an indicator, of the position of the user uploaded value, among the reference. Figure 5 demonstrates a screenshot of the output of the first few columns from the R-package PredictAP. The first four columns represent the data in the exact format as imported by the user. Columns 5–10 shows the results as provided by PredictAP for the users to evaluate the gene expressions predicted by PrediXcan. The mean and standard deviation values of each gene in the reference set and the number of variants can help users decipher the reliability and impact of population diversity holistically. For example, if users input the predicted gene expression values from subcutaneous adipose tissue, the algorithm first matches them to the reference genes of the corresponding tissue. Next, the percentile ranks for each predicted gene expression value is calculated among the reference. The number of variants used, average expression, and standard deviation are also matched to the corresponding gene. The last two columns provide whether the difference between the EAS and European populations for the same gene are significant (Sig.) or not (Nonsig.), and the mean differences between predicted gene expression by using Predixcan and EAS Reference, respectively. Output data from all genes are presented for a given subject followed by all genes from the next subject, and so on. Since most subjects in the reference panel have gene expression levels centered around a percentile rank of 50, the deviations from this value suggest greater differences in gene expression compared to the normal. For instance, in Fig. 5, the Kolmogorov–Smirnov test indicates that gene ENSG00000000457.13 has significant population differences, exhibiting ~ 85.7% lower expression compared to the European population. Moreover, the percentile rank of 2.4 suggests relatively lower gene expression levels in the EAS reference group. Despite determining whether these differences would be risk or protective factors requires further analysis of specific phenotypes, our approach could mitigate the impact of population difference when prioritizing the targeted genes.

An additional evaluation was also conducted using Bonferroni corrected P-values for the readers to establish the importance of an EAS specific reference panel (threshold is set by dividing 1 by the number of genes predictable in that tissue (e.g., for the Liver, which can predict 7969 genes, the threshold would be 1/7969). The results (Table S3) demonstrated that the over 80% of genes for each tissue showed significant differences between European and EAS populations. Furthermore, Fig. S5 demonstrates distribution plots for a gene with expression significantly different and another gene not significantly different between European and EAS, respectively.

### Evaluation of PrediXcan predictions for real patient data using PredictAP

Correlation analysis between inverse normalized real gene expression values, GE_Real_(normalized) and predicted gene expression values by PrediXcan (GE_Predixcan_) utilizing SNP array, both from female non-smoking lung cancer patients (GSE33356) reveals results that were severely biased (Table S4). The correlation values were heavily clustered towards negative values although the maximum correlation value was reported as 0.780. One of the possible reasons for such bias could be the low sample size of the lung cancer dataset which is almost a quarter of the size of the PrediXcan training data. Such low sample size could be responsible for hindering a stable normalization. Based on GTEx’s sample size (https://gtexportal.org/home/tissueSummaryPage) of ~1000 donors and other prior studies on gene expression prediction, to get a stable inverse normalization while working on prediction using PrediXcan it would be safer to use a sample size of ~1000 [15, 19, 20]. Additionally, possible presence of unknown artifacts or processing steps could also be responsible for such bias. All of the above indicated a challenge towards conducting external validation. Hence, GE_PrediXcan_ using SNPs from both the lung cancer and gastric cancer datasets were evaluated against PredictAP (Table S5 and S6). For both the tables S5 and S6, column ***Gene*** provides the Gene IDs; ***Ref.Mean*** and ***Ref. Sd.*** provide the mean and standard deviation from all samples in PredictAP, respectively; ***Data.Mean*** and ***Data.Sd*** are the mean and standard deviation, of all samples in the datasets (lung or blood), respectively; ***Abs.Diff***. gives the absolute difference of the Data.Mean from the Ref.Mean; ***Ratio.Diff*** column is the ratio of Abs.Diff and Ref.Mean; ***Data.SNP.Num*** denotes the number of SNPs that we used from the lung or blood data to run PrediXcan; while column ***Predixcan_SNP.Num*** denotes the necessary number of SNPs that PrediXcan utilized to provide GE_Predixcan_ from each datasets; ***SNP.Ratio*** provides the ratio of Data.SNP.Num and Predixcan_SNP.Num. It is to be noted that if Data.SNP.Num < Predixcan_SNP.Num then the missing SNPs will be replaced by the references’ wild type allele to be used by PrediXcan for prediction. Finally, the last two columns provides an evaluation of the GE_Predixcan_ with reference to PredictAP; where column ***GE.Match.Ref.Num*** denotes the number of samples from the datasets (lung or blood) that has GE_Predixcan_ overlapping with the CI from PredictAP; and column ***GE.Match.Ref.Ratio*** provides the corresponding proportion from among all samples in the respective datasets. Based on both the tables, users conducting studies on EAS populations, should adopt caution for the genes whose PrediXcan predictions demonstrate dramatic deviation from PredictAP, for instance *ENSG00000000971.15* (Table S5) and *ENSG00000003137.8* (Table S6) because they may have ethnic based effect on their gene expression. On the other hand, for the genes showing good concordance between PredictAP and PrediXcan, (example: *ENSG00000000938.12* from Table S5, and *ENSG00000000419.12* from Table S6), users can continue to analyze them safely based on PrediXcan reports. A detailed tutorial using the lung cancer dataset (GSE33356) (**PredictAP tutorial.pdf**) has been additionally provided for clarity.

## Discussion

In this study, we evaluated the impact of the population-level genetic differences on the results of PrediXcan in two ways. First, the weights for each variant from the gene expression model were utilized to simulate the variation of the predicted gene expression values in the presence of population-differentiated variants present in the model. Second, the parameter of proportion of population differentiated variants was used to control the degree of population diversity. Utilizing the dosage for each variant generated from the corresponding MAF, the predicted gene expression was compared. As expected, a high impact of each of the above parameters on gene expression were observed, among which weight was the most effective one suggesting that it is essential that population specific differences be incorporated while conducting gene expression prediction. Therefore, we proposed a method that leveraged allele frequency information from EAS ancestry to develop a reference that incorporates population diversity. Because the data used to develop and train PrediXcan were collected from subjects of European ancestry, the predictions of gene expression in subjects of EAS ancestry would be incorrect due to different allele frequency patterns. The percentile ranks from the R-package ‘PredictAP’ developed in this study can be used to adjust the weighting for predicted gene expression in different ancestries, mitigating the negative impacts of population differences.

Other popular methods such as FUSION and S-PrediXcan are very similar to PrediXcan, in how they function, however, each of them demonstrate distinct features, as well. Both FUSION and S-PrediXcan methods allow users to use GWAS summary statistic to test the association between gene and trait whereas PrediXcan applies individual-level genotype data. However, the reference for all the three methods are derived from European population, which potentially leads to eluding the effect of the under-represented populations. For example, as FUSION considers GWAS summary statistic and heritability, it may get biased conclusions when heritability varies across populations. On the other hand, PredictAP can provide insight into genetic differences between European and EAS populations. Our proposed approach can be viewed as an auxiliary tool for PrediXcan when using the genotype data of EAS subjects. In practice, as PrediXcan requests individual-level data, the information for each variant may not be complete due to the limitations of platform design or the quality of imputation with a small sample size. Our reference panel can help bridge the gap from genotype to gene expression by providing a reference value for each predicted gene expression. On the other hand, the reference values for each gene can be taken as normal expression, which means this information can be integrated with other data to conduct additional analysis (e.g., pathway analysis). For example, in differential gene expression analysis, our reference panel can be applied as a control group for comparison to external gene expression data.

An interesting study by Taylor et al. focused on varied sources of gene expression variation in a globally diverse human cohort where they identified two population-specific QTLs: (i) frequency differentiated QTLs (fd-QTLs) which has different allele frequencies across continental groups and (ii) heterogeneous effect QTLs (he-QTLs) that demonstrate heterogeneity of effect sizes heterogeneity between continental groups [21]. The population differentiated eQTLs in our study are the fd-QTLs that were referred to in their study and their conclusions reiterated our findings where they concluded that fd-QTLs are responsible for gene expression differences across populations. However, for he-QTLs they observed that the magnitude and direction of the causal ones are consistent across populations and that ‘population-specific’ effects that were observed in prior studies were due to low-resolution or other independent eQTLs of the same genes that went undetected. The concept of he-QTLs is interesting and warrants further exploration, however was not under the scope of the current study.

There were several limitations in this study. First, in order to utilize our R-Package with PrediXcan, individual-level genotyping data are needed. However, despite the fact that several summary statistic methods have been developed for the TWAS field, including FUSION [22] and S-PrediXcan [17], population diversity remains a concern. This is because the expression profiles of diverse populations differ, and the subjects that comprised the training data for both methods were mostly of European ancestry. Second, our proposed method aims to provide insight into genetic differences between European and EAS populations. To resolve the population diversity issue comprehensively, large-scale sequencing data that collects both transcriptome and genome profiles from EAS ancestry to develop the gene expression prediction model would be needed. However, accessing multi-omic data remains a challenging task due to the cost of measuring expression and subject availability. Alternatively, transfer learning [23–25] could potentially boost the prediction performance by transferring gene expression prediction-related knowledge from the GTEx dataset to improve the prediction performance for a relatively small sample of Asian-based multi-omics data. Recently, a transfer learning scheme was applied to biomedical data, showing a promising improvement [26–28]. Finally, the lack of experimental validation as a result of the bias caused by the low sample size of the patient datasets and inherent normalization within PrediXcan is another limitation of the study. However, this was compensated by constructing CI s and sets of summary parameters in PredictAP based on empirical distribution of per gene per tissue. Often, the approximation provided by empirical distributions are sufficient and efficient ways to understand how much a statistic can vary and empirical distributions are often used to understand the properties of a statistic. Furthermore, experimental data were used to demonstrate how to evaluate gene expression predictions against PredictAP.

## Conclusion

We demonstrated in this study that the performance of gene expression prediction using PrediXcan would be affected by population diversity. Also, the allele frequency pattern of cis-variants is indeed different between subjects of European and EAS ancestry. Caution is required when applying PrediXcan to data from subjects of EAS ancestry. Here, we developed a gene expression reference for EAS data as an auxiliary tool to check the reliability of the gene expression prediction. In future studies, our reference may have further applications that utilize the percentile rank as an adjusted weight to determine the effect size of EAS ancestry.

**Key Points**

- The stability of predicted gene expression in PrediXcan in subjects of East Asian (EAS) ancestry remains unclear.
- We demonstrated the impact of population diversity through simulation, showing that the weight value of the variant is the primary factor in predicting gene expression.
- Approximately 70% of variants have significantly different allele frequency patterns between European and EAS ancestry by the proportion test.
- We proposed a gene expression reference profile for EAS ancestry to provide insight into the reliability of gene expression predictions by PrediXcan.

## Supplementary Material

Supplementary_File_1_revision2_final_0831_bbae549

Table_S5_Lungcancer_GSE33356_bbae549

Table_S6_BloodDNA_GSE26853_bbae549

PreditAP_turorial0831_bbae549

## Data Availability

The PrediXcan models are available at https://predictdb.org/ and gnomAD allele frequency data is available at https://gnomad.broadinstitute.org/downloads. The algorithm of the package is illustrated in Fig. 3 and the package is available on the following link: https://www.space.ntu.edu.tw/navigate/a/#/s/53A4746DCB2C4B1E9E7E37AFEB17892A6BL.
