# Supplementary material for: Cross-population enhancement of PrediXcan predictions with a gnomAD-based east Asian reference framework
Source: Brief Bioinform. 2024 Oct 23;25(6):bbae549. doi: 10.1093/bib/bbae549 (PMC11497844; doi:10.1093/bib/bbae549)
Supplement: Supplementary_File_1_revision2_final_0831_bbae549 [file supplementary_file_1_revision2_final_0831_bbae549.docx]

**Supplementary Materials**

**Cross-Population Enhancement of PrediXcan Predictions with a gnomAD-based East Asian Reference Framework**

Han-Ching Chan^1^, Amrita Chattopadhyay^1^, Tzu-Pin Lu^1,2^*

^1^ Institute of Epidemiology and Preventive Medicine, Department of Public Health, National Taiwan University, Taipei, Taiwan

^2^ Institute of Health Data Analytics and Statistics, Department of Public Health, National Taiwan University, Taipei, Taiwan

* Corresponding author:

Tzu-Pin Lu, Ph.D.

Room 518, No. 17, Xu-Zhou Road, Taipei 10055, Taiwan

Phone: +886-2- 33668042

E-mail: [tplu@ntu.edu.tw](mailto:tplu@ntu.edu.tw)

**Han-Ching Chan** is a PhD candidate in the Institute of Epidemiology and Preventive Medicine, National Taiwan University, Taipei, Taiwan.

**Amrita Chattopadhyay** is an Assistant Professor at the Institute of Epidemiology and Preventive Medicine, National Taiwan University, Taipei, Taiwan

**Tzu-Pin Lu** is a Professor and Director at the Institute of Health Data analytics and Statistics, National Taiwan University, Taipei, Taiwan.

**
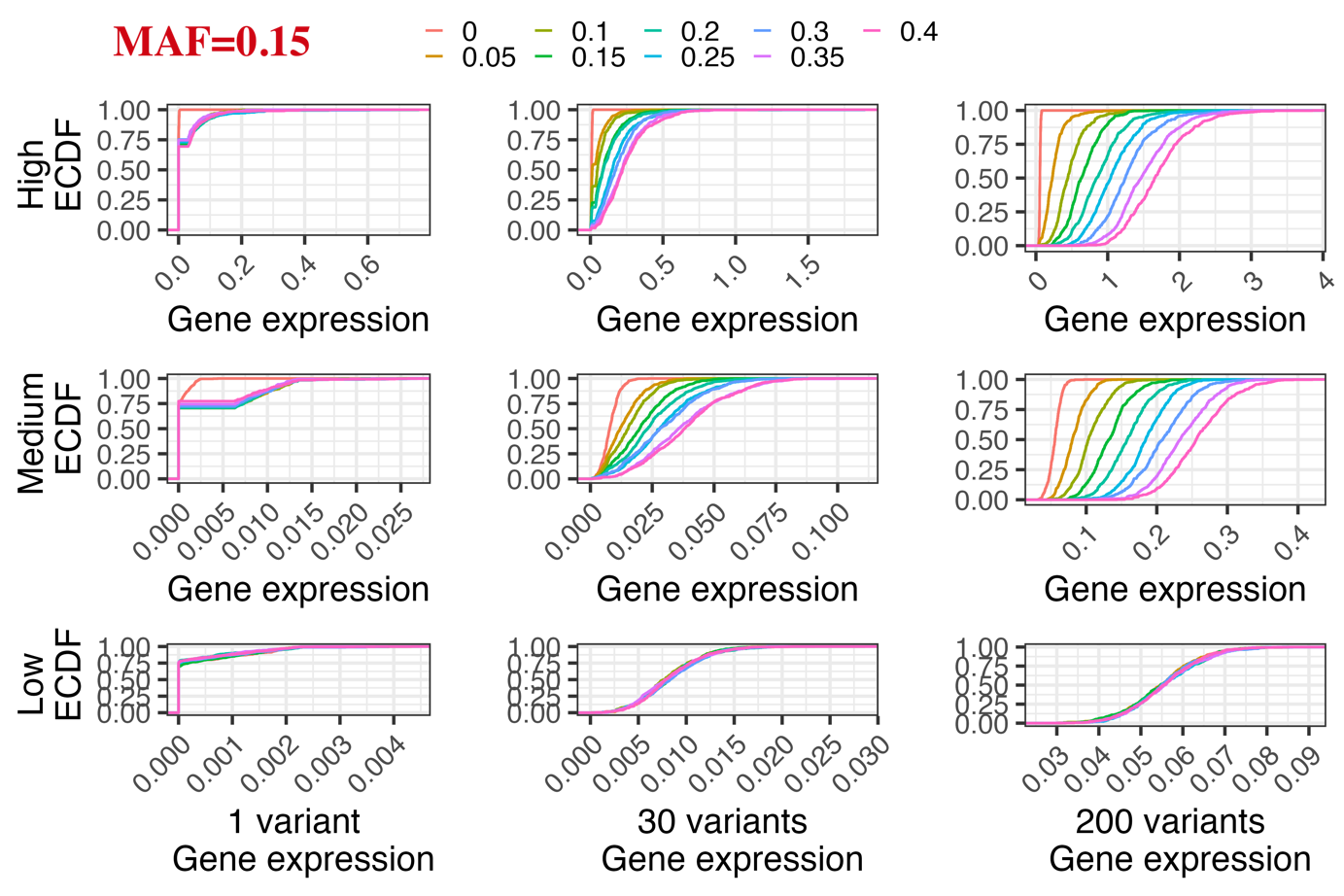
**

Figure S1. The ECDF curve of simulation results for MAF equal to 0.15

**
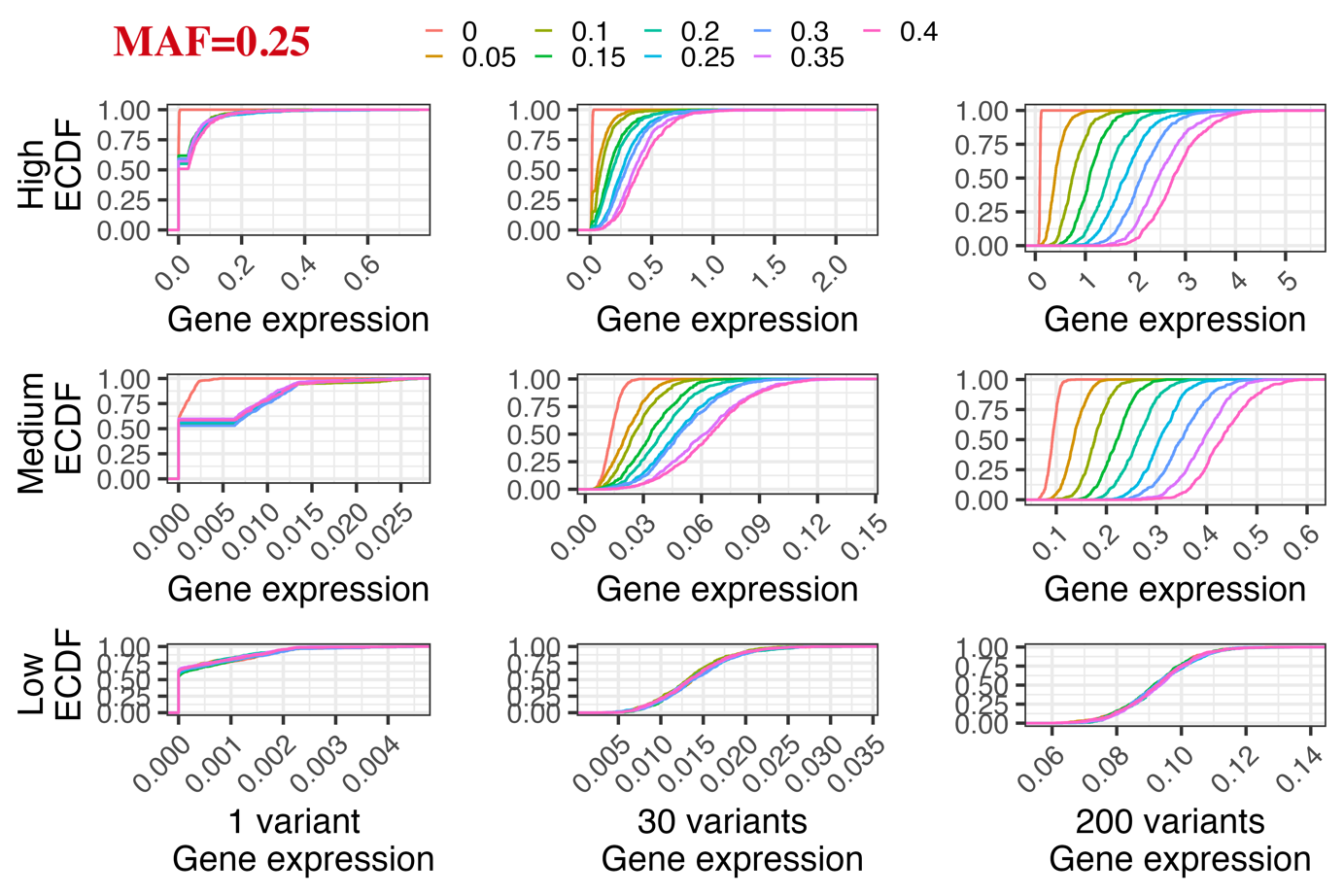
**

Figure S2. The ECDF curve of simulation results for MAF equal to 0.25

**
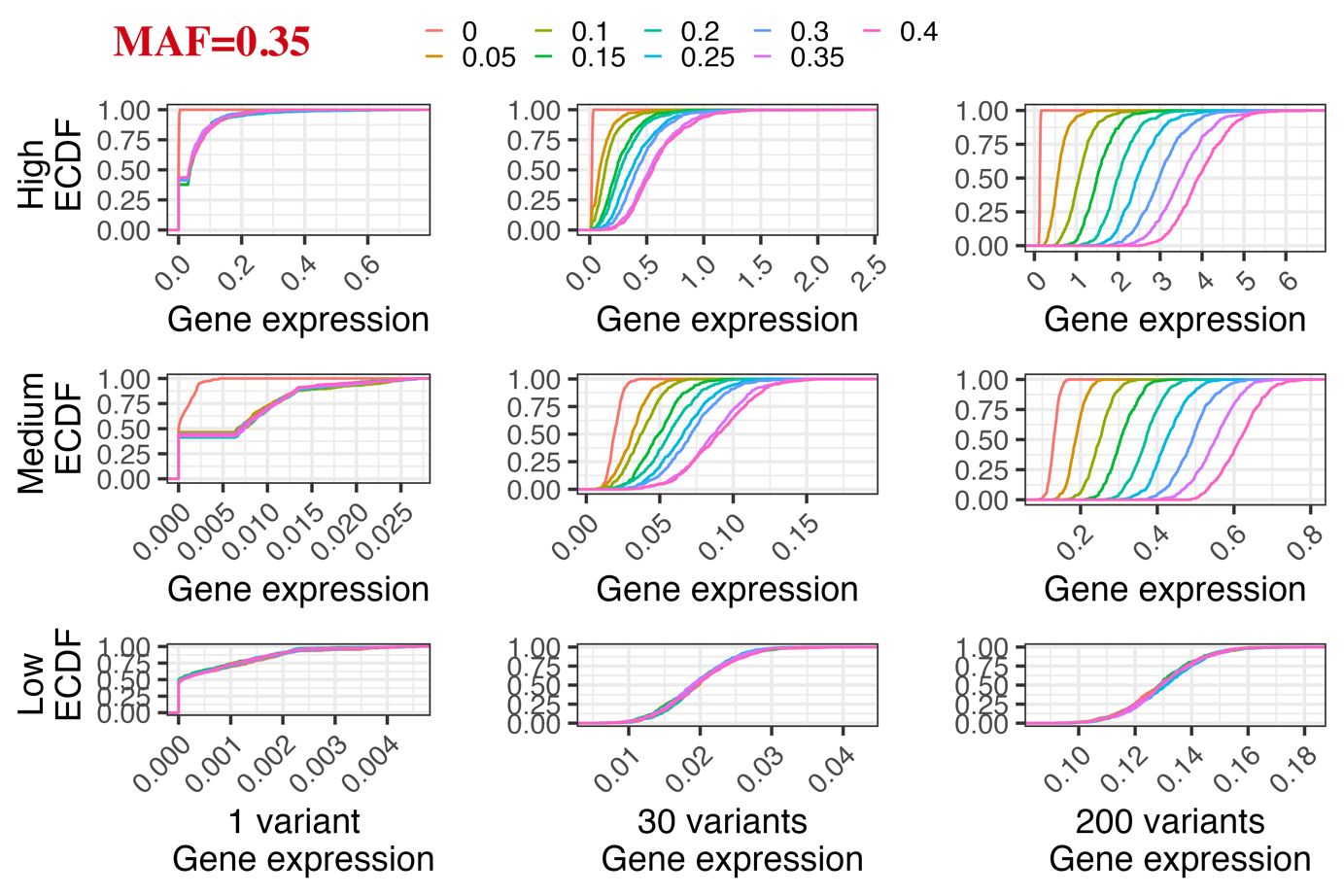
**

Figure S3. The ECDF curve of simulation results for MAF equal to 0.35

**
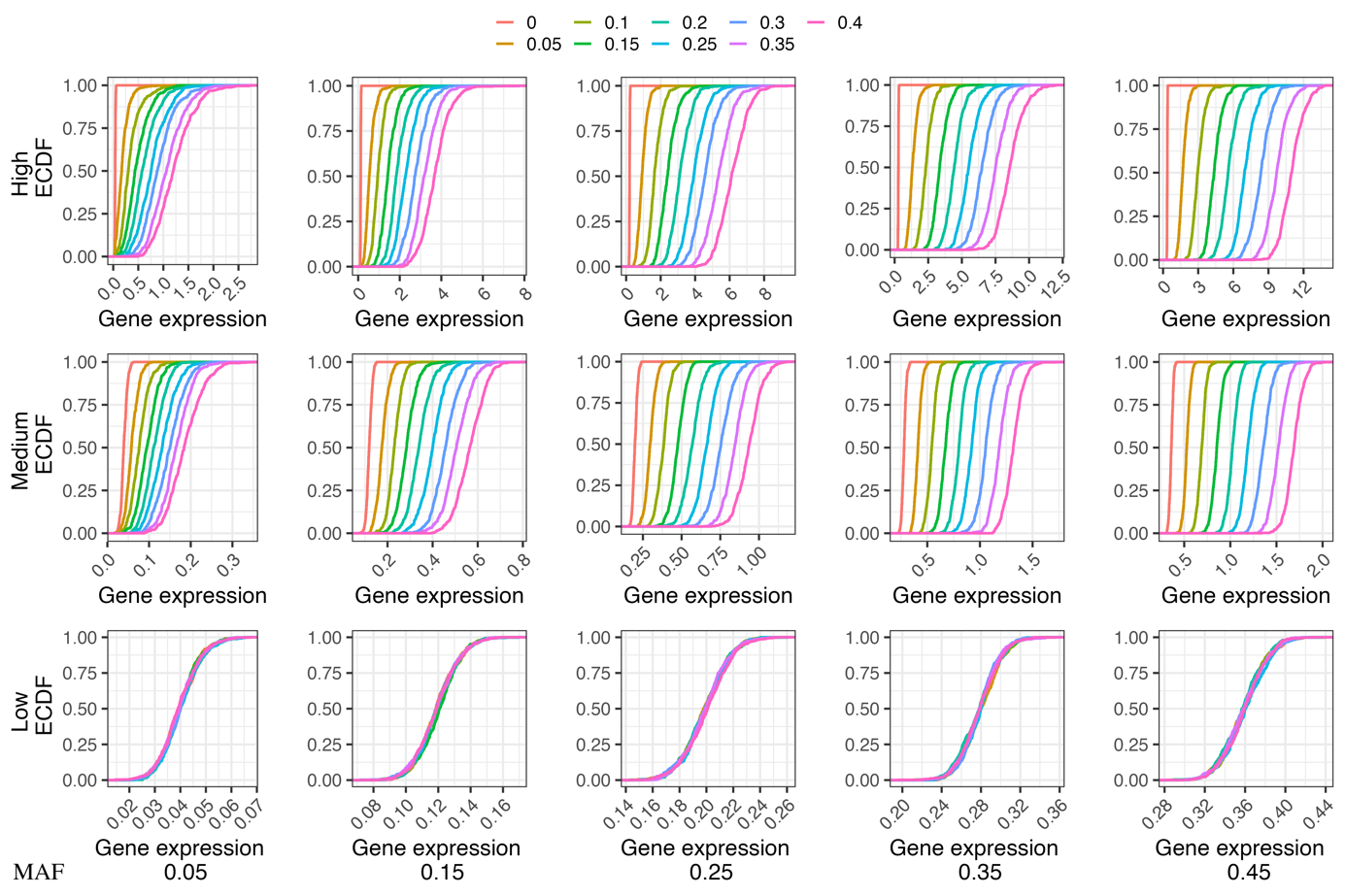
**

Figure S4. The ECDF curve of simulation results under 430 variants. The outer X-axis represents the MAF, and the outer Y-axis represents the weight level of population-differentiated variants. Each line shows the ECDF curve of predicted gene expression. The colors listed in the legend explain the proportion of population-differentiated variants. The closer lines mean the distribution of predicted gene expression was more similar.

**
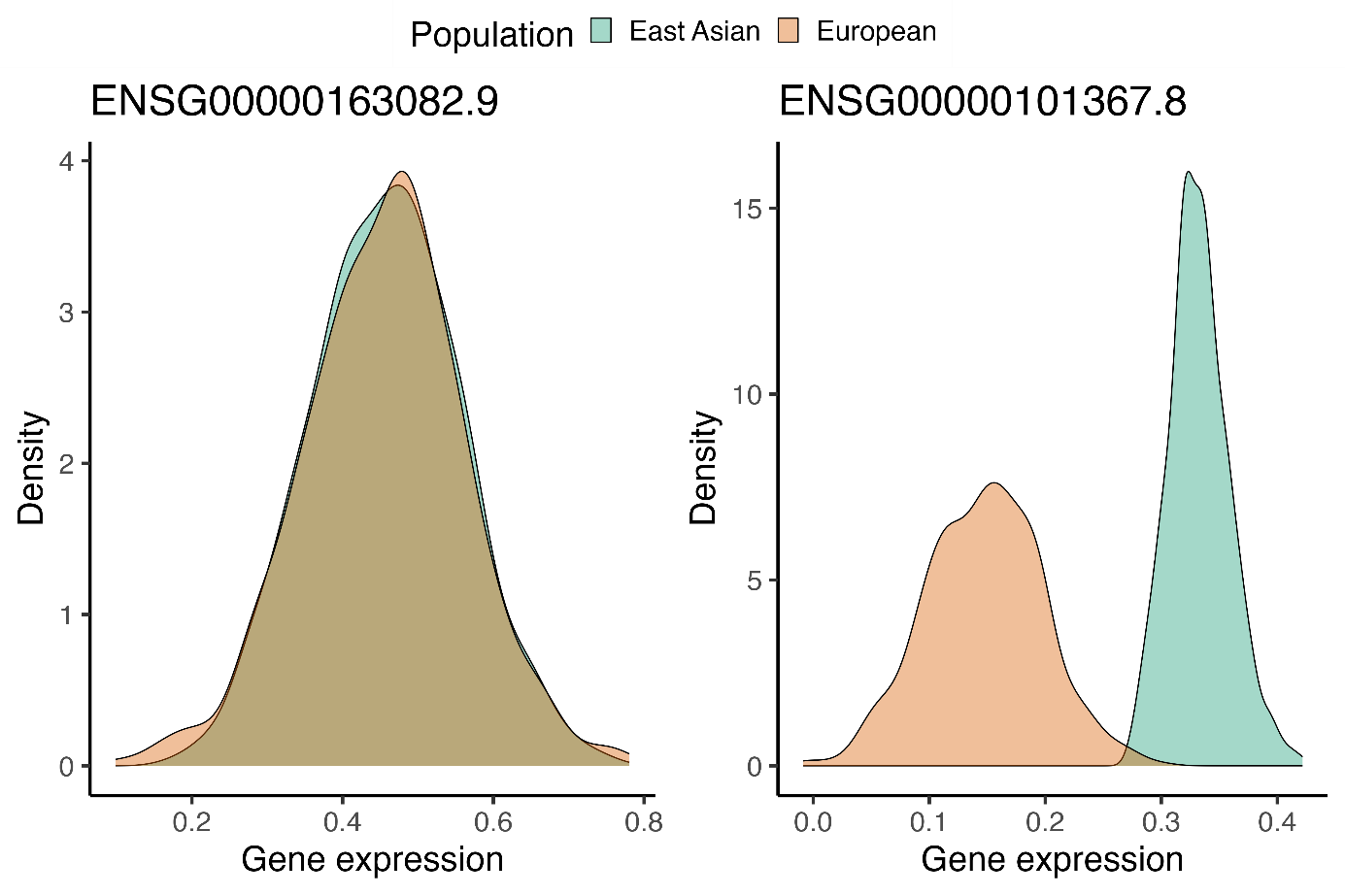
**

Figure S5. Distribution plots for two genes. Left: overlapping densities with not significant difference in gene expression between East Asian and European. Right: not overlapping densities with significant difference of gene expression between East Asian and Europeans.

Table S1. Summary of gene expression model for 49 tissues in GTEx v8 elastic net models.

| **No.** | Gene expression models | Number of genes/SNPs | **No.** | Gene expression models | Number of genes/SNPs |
| --- | --- | --- | --- | --- | --- |
| **1** | Adipose_Subcutaneous | 8,650/214,645 | **26** | Esophagus_Mucosa | 8,521/214,022 |
| **2** | Adipose_Visceral_Omentum | 7,340/178,760 | **27** | Esophagus_Muscularis | 8,231/207,468 |
| **3** | Adrenal_Gland | 4,843/132,957 | **28** | Heart_Atrial_Appendage | 6,641/170,801 |
| **4** | Artery_Aorta | 7,599/196,728 | **29** | Heart_Left_Ventricle | 6,013/150,861 |
| **5** | Artery_Coronary | 4,046/113,130 | **30** | Kidney_Cortex | 1,642/60,744 |
| **6** | Artery_Tibial | 8,615/221,893 | **31** | Liver | 3,773/105,390 |
| **7** | Brain_Amygdala | 2,787/86,805 | **32** | Lung | 7,969/194,164 |
| **8** | Brain_Anterior_cingulate_cortex_BA24 | 3,544/106,328 | **33** | Minor_Salivary_Gland | 2,916/91,751 |
| **9** | Brain_Caudate_basal_ganglia | 5,004/144,730 | **34** | Muscle_Skeletal | 7,583/186,517 |
| **10** | Brain_Cerebellar_Hemisphere | 5,753/173,681 | **35** | Nerve_Tibial | 10,012/259,465 |
| **11** | Brain_Cerebellum | 6,794/196,723 | **36** | Ovary | 3,587/107,125 |
| **12** | Brain_Cortex | 5,500/157,608 | **37** | Pancreas | 5,896/158,802 |
| **13** | Brain_Frontal_Cortex_BA9 | 4,563/132,061 | **38** | Pituitary | 5,688/152,008 |
| **14** | Brain_Hippocampus | 3,688/107,480 | **39** | Prostate | 4,302/119,076 |
| **15** | Brain_Hypothalamus | 3,652/105,843 | **40** | Skin_Not_Sun_Exposed_Suprapubic | 8,650/216,250 |
| **16** | Brain_Nucleus_accumbens_basal_ganglia | 4,851/137,024 | **41** | Skin_Sun_Exposed_Lower_leg | 9,299/230,117 |
| **17** | Brain_Putamen_basal_ganglia | 4,436/129,768 | **42** | Small_Intestine_Terminal_Ileum | 3,670/107,287 |
| **18** | Brain_Spinal_cord_cervical_c-1 | 3,250/104,122 | **43** | Spleen | 5,774/161,387 |
| **19** | Brain_Substantia_nigra | 2,559/83,791 | **44** | Stomach | 5,156/133,323 |
| **20** | Breast_Mammary_Tissue | 6,461/157,911 | **45** | Testis | 9,978/264,789 |
| **21** | Cells_Cultured_fibroblasts | 8,933/236,279 | **46** | Thyroid | 9,652/243,941 |
| **22** | Cells_EBV-transformed_lymphocytes | 2,904/93,235 | **47** | Uterus | 2,541/80,231 |
| **23** | Colon_Sigmoid | 6,173/159,704 | **48** | Vagina | 2,562/77,872 |
| **24** | Colon_Transverse | 6,304/159,506 | **49** | Whole_Blood | 7,252/177,016 |
| **25** | Esophagus_Gastroesophageal_Junction | 6,291/164,531 |  |  |  |

Table S2. For each simulation combination, we calculated the average absolute difference and ratio of gene expression to represent the impact of population disparity. The baseline for calculation was set as zero percent of population-differentiated variants.

* >99.0000: large number due to the calculation with a small value in denominator.

| MAF | No. of Variants | Percent of significant | Weight level | Absolute difference | Ratio* |
| --- | --- | --- | --- | --- | --- |
| 0.05 | 1 | 0.05 | High | 0.0104 | 9.0000 |
| 0.05 | 1 | 0.1 | High | 0.0090 | 30.0000 |
| 0.05 | 1 | 0.15 | High | 0.0072 | 16.0000 |
| 0.05 | 1 | 0.2 | High | 0.0068 | >99.0000 |
| 0.05 | 1 | 0.25 | High | 0.0092 | 10.0000 |
| 0.05 | 1 | 0.3 | High | 0.0181 | >99.0000 |
| 0.05 | 1 | 0.35 | High | 0.0134 | >99.0000 |
| 0.05 | 1 | 0.4 | High | 0.0038 | 4.0000 |
| 0.05 | 30 | 0.05 | High | 0.0146 | >99.0000 |
| 0.05 | 30 | 0.1 | High | 0.0221 | >99.0000 |
| 0.05 | 30 | 0.15 | High | 0.0400 | >99.0000 |
| 0.05 | 30 | 0.2 | High | 0.0385 | >99.0000 |
| 0.05 | 30 | 0.25 | High | 0.0587 | >99.0000 |
| 0.05 | 30 | 0.3 | High | 0.0612 | >99.0000 |
| 0.05 | 30 | 0.35 | High | 0.0755 | >99.0000 |
| 0.05 | 30 | 0.4 | High | 0.0857 | >99.0000 |
| 0.05 | 200 | 0.05 | High | 0.0613 | >99.0000 |
| 0.05 | 200 | 0.1 | High | 0.1370 | >99.0000 |
| 0.05 | 200 | 0.15 | High | 0.1937 | >99.0000 |
| 0.05 | 200 | 0.2 | High | 0.2701 | >99.0000 |
| 0.05 | 200 | 0.25 | High | 0.3391 | >99.0000 |
| 0.05 | 200 | 0.3 | High | 0.4016 | >99.0000 |
| 0.05 | 200 | 0.35 | High | 0.5026 | >99.0000 |
| 0.05 | 200 | 0.4 | High | 0.5404 | >99.0000 |
| 0.05 | 430 | 0.05 | High | 0.1719 | 4.1522 |
| 0.05 | 430 | 0.1 | High | 0.3464 | 8.3110 |
| 0.05 | 430 | 0.15 | High | 0.5064 | 12.3333 |
| 0.05 | 430 | 0.2 | High | 0.6624 | 15.9565 |
| 0.05 | 430 | 0.25 | High | 0.8002 | 19.1481 |
| 0.05 | 430 | 0.3 | High | 0.9896 | 23.7171 |
| 0.05 | 430 | 0.35 | High | 1.1211 | 26.9010 |
| 0.05 | 430 | 0.4 | High | 1.2946 | 31.1415 |
| 0.05 | 1 | 0.05 | Medium | 0.0016 | >99.0000 |
| 0.05 | 1 | 0.1 | Medium | 0.0022 | >99.0000 |
| 0.05 | 1 | 0.15 | Medium | 0.0019 | >99.0000 |
| 0.05 | 1 | 0.2 | Medium | 0.0014 | 2.0000 |
| 0.05 | 1 | 0.25 | Medium | 0.0015 | 8.0000 |
| 0.05 | 1 | 0.3 | Medium | 0.0014 | >99.0000 |
| 0.05 | 1 | 0.35 | Medium | 0.0014 | 10.0000 |
| 0.05 | 1 | 0.4 | Medium | 0.0020 | >99.0000 |
| 0.05 | 30 | 0.05 | Medium | 0.0035 | >99.0000 |
| 0.05 | 30 | 0.1 | Medium | 0.0044 | >99.0000 |
| 0.05 | 30 | 0.15 | Medium | 0.0058 | >99.0000 |
| 0.05 | 30 | 0.2 | Medium | 0.0067 | >99.0000 |
| 0.05 | 30 | 0.25 | Medium | 0.0080 | >99.0000 |
| 0.05 | 30 | 0.3 | Medium | 0.0082 | >99.0000 |
| 0.05 | 30 | 0.35 | Medium | 0.0112 | >99.0000 |
| 0.05 | 30 | 0.4 | Medium | 0.0109 | >99.0000 |
| 0.05 | 200 | 0.05 | Medium | 0.0112 | 0.7132 |
| 0.05 | 200 | 0.1 | Medium | 0.0184 | 1.1624 |
| 0.05 | 200 | 0.15 | Medium | 0.0252 | 1.5744 |
| 0.05 | 200 | 0.2 | Medium | 0.0350 | 2.1510 |
| 0.05 | 200 | 0.25 | Medium | 0.0437 | 2.6954 |
| 0.05 | 200 | 0.3 | Medium | 0.0518 | 3.1768 |
| 0.05 | 200 | 0.35 | Medium | 0.0603 | 3.6753 |
| 0.05 | 200 | 0.4 | Medium | 0.0683 | 4.1681 |
| 0.05 | 430 | 0.05 | Medium | 0.0219 | 0.5473 |
| 0.05 | 430 | 0.1 | Medium | 0.0419 | 1.0308 |
| 0.05 | 430 | 0.15 | Medium | 0.0623 | 1.5183 |
| 0.05 | 430 | 0.2 | Medium | 0.0793 | 1.9309 |
| 0.05 | 430 | 0.25 | Medium | 0.1015 | 2.4609 |
| 0.05 | 430 | 0.3 | Medium | 0.1211 | 2.9224 |
| 0.05 | 430 | 0.35 | Medium | 0.1442 | 3.4830 |
| 0.05 | 430 | 0.4 | Medium | 0.1648 | 3.9421 |
| 0.05 | 1 | 0.05 | Low | 0.0011 | 1.1794 |
| 0.05 | 1 | 0.1 | Low | 0.0011 | 0.9789 |
| 0.05 | 1 | 0.15 | Low | 0.0011 | >99.0000 |
| 0.05 | 1 | 0.2 | Low | 0.0011 | 0.9871 |
| 0.05 | 1 | 0.25 | Low | 0.0011 | 0.9715 |
| 0.05 | 1 | 0.3 | Low | 0.0010 | 0.9670 |
| 0.05 | 1 | 0.35 | Low | 0.0011 | 0.9741 |
| 0.05 | 1 | 0.4 | Low | 0.0010 | 0.9864 |
| 0.05 | 30 | 0.05 | Low | 0.0021 | >99.0000 |
| 0.05 | 30 | 0.1 | Low | 0.0021 | >99.0000 |
| 0.05 | 30 | 0.15 | Low | 0.0021 | >99.0000 |
| 0.05 | 30 | 0.2 | Low | 0.0023 | >99.0000 |
| 0.05 | 30 | 0.25 | Low | 0.0023 | >99.0000 |
| 0.05 | 30 | 0.3 | Low | 0.0021 | >99.0000 |
| 0.05 | 30 | 0.35 | Low | 0.0022 | >99.0000 |
| 0.05 | 30 | 0.4 | Low | 0.0022 | >99.0000 |
| 0.05 | 200 | 0.05 | Low | 0.0058 | 0.3524 |
| 0.05 | 200 | 0.1 | Low | 0.0063 | 0.3872 |
| 0.05 | 200 | 0.15 | Low | 0.0059 | 0.3580 |
| 0.05 | 200 | 0.2 | Low | 0.0060 | 0.3762 |
| 0.05 | 200 | 0.25 | Low | 0.0059 | 0.3598 |
| 0.05 | 200 | 0.3 | Low | 0.0061 | 0.3675 |
| 0.05 | 200 | 0.35 | Low | 0.0060 | 0.3638 |
| 0.05 | 200 | 0.4 | Low | 0.0059 | 0.3633 |
| 0.05 | 430 | 0.05 | Low | 0.0091 | 0.2209 |
| 0.05 | 430 | 0.1 | Low | 0.0094 | 0.2235 |
| 0.05 | 430 | 0.15 | Low | 0.0093 | 0.2246 |
| 0.05 | 430 | 0.2 | Low | 0.0095 | 0.2272 |
| 0.05 | 430 | 0.25 | Low | 0.0090 | 0.2130 |
| 0.05 | 430 | 0.3 | Low | 0.0094 | 0.2276 |
| 0.05 | 430 | 0.35 | Low | 0.0097 | 0.2347 |
| 0.05 | 430 | 0.4 | Low | 0.0094 | 0.2225 |
| 0.15 | 1 | 0.05 | High | 0.0196 | >99.0000 |
| 0.15 | 1 | 0.1 | High | 0.0185 | >99.0000 |
| 0.15 | 1 | 0.15 | High | 0.0282 | >99.0000 |
| 0.15 | 1 | 0.2 | High | 0.0240 | >99.0000 |
| 0.15 | 1 | 0.25 | High | 0.0272 | >99.0000 |
| 0.15 | 1 | 0.3 | High | 0.0261 | >99.0000 |
| 0.15 | 1 | 0.35 | High | 0.0173 | >99.0000 |
| 0.15 | 1 | 0.4 | High | 0.0237 | >99.0000 |
| 0.15 | 30 | 0.05 | High | 0.0399 | 6.1278 |
| 0.15 | 30 | 0.1 | High | 0.0608 | 9.8692 |
| 0.15 | 30 | 0.15 | High | 0.1046 | 19.5710 |
| 0.15 | 30 | 0.2 | High | 0.1135 | 18.9525 |
| 0.15 | 30 | 0.25 | High | 0.1726 | 28.1316 |
| 0.15 | 30 | 0.3 | High | 0.1863 | 30.1710 |
| 0.15 | 30 | 0.35 | High | 0.2273 | 40.0774 |
| 0.15 | 30 | 0.4 | High | 0.2418 | 38.7596 |
| 0.15 | 200 | 0.05 | High | 0.1951 | 3.6277 |
| 0.15 | 200 | 0.1 | High | 0.4220 | 7.7838 |
| 0.15 | 200 | 0.15 | High | 0.6093 | 11.2393 |
| 0.15 | 200 | 0.2 | High | 0.8266 | 15.2155 |
| 0.15 | 200 | 0.25 | High | 1.0187 | 18.7785 |
| 0.15 | 200 | 0.3 | High | 1.2273 | 22.6330 |
| 0.15 | 200 | 0.35 | High | 1.4572 | 26.9003 |
| 0.15 | 200 | 0.4 | High | 1.6464 | 30.3621 |
| 0.15 | 430 | 0.05 | High | 0.5099 | 3.9575 |
| 0.15 | 430 | 0.1 | High | 0.9999 | 7.7700 |
| 0.15 | 430 | 0.15 | High | 1.4628 | 11.3587 |
| 0.15 | 430 | 0.2 | High | 1.9360 | 15.0275 |
| 0.15 | 430 | 0.25 | High | 2.4319 | 18.8712 |
| 0.15 | 430 | 0.3 | High | 2.9576 | 22.9349 |
| 0.15 | 430 | 0.35 | High | 3.3911 | 26.2914 |
| 0.15 | 430 | 0.4 | High | 3.8303 | 29.7389 |
| 0.15 | 1 | 0.05 | Medium | 0.0031 | >99.0000 |
| 0.15 | 1 | 0.1 | Medium | 0.0029 | >99.0000 |
| 0.15 | 1 | 0.15 | Medium | 0.0032 | >99.0000 |
| 0.15 | 1 | 0.2 | Medium | 0.0035 | >99.0000 |
| 0.15 | 1 | 0.25 | Medium | 0.0028 | >99.0000 |
| 0.15 | 1 | 0.3 | Medium | 0.0031 | >99.0000 |
| 0.15 | 1 | 0.35 | Medium | 0.0030 | >99.0000 |
| 0.15 | 1 | 0.4 | Medium | 0.0036 | >99.0000 |
| 0.15 | 30 | 0.05 | Medium | 0.0073 | 1.6405 |
| 0.15 | 30 | 0.1 | Medium | 0.0092 | 1.7806 |
| 0.15 | 30 | 0.15 | Medium | 0.0138 | 2.7943 |
| 0.15 | 30 | 0.2 | Medium | 0.0173 | 3.3364 |
| 0.15 | 30 | 0.25 | Medium | 0.0218 | 4.3483 |
| 0.15 | 30 | 0.3 | Medium | 0.0223 | 4.0758 |
| 0.15 | 30 | 0.35 | Medium | 0.0299 | 5.6200 |
| 0.15 | 30 | 0.4 | Medium | 0.0310 | 5.5088 |
| 0.15 | 200 | 0.05 | Medium | 0.0283 | 0.5502 |
| 0.15 | 200 | 0.1 | Medium | 0.0517 | 0.9864 |
| 0.15 | 200 | 0.15 | Medium | 0.0778 | 1.4740 |
| 0.15 | 200 | 0.2 | Medium | 0.1038 | 1.9575 |
| 0.15 | 200 | 0.25 | Medium | 0.1299 | 2.4397 |
| 0.15 | 200 | 0.3 | Medium | 0.1535 | 2.8780 |
| 0.15 | 200 | 0.35 | Medium | 0.1800 | 3.3735 |
| 0.15 | 200 | 0.4 | Medium | 0.2033 | 3.8050 |
| 0.15 | 430 | 0.05 | Medium | 0.0619 | 0.4903 |
| 0.15 | 430 | 0.1 | Medium | 0.1219 | 0.9572 |
| 0.15 | 430 | 0.15 | Medium | 0.1843 | 1.4444 |
| 0.15 | 430 | 0.2 | Medium | 0.2428 | 1.8967 |
| 0.15 | 430 | 0.25 | Medium | 0.3041 | 2.3743 |
| 0.15 | 430 | 0.3 | Medium | 0.3633 | 2.8328 |
| 0.15 | 430 | 0.35 | Medium | 0.4286 | 3.3451 |
| 0.15 | 430 | 0.4 | Medium | 0.4932 | 3.8450 |
| 0.15 | 1 | 0.05 | Low | 0.0011 | >99.0000 |
| 0.15 | 1 | 0.1 | Low | 0.0010 | >99.0000 |
| 0.15 | 1 | 0.15 | Low | 0.0010 | >99.0000 |
| 0.15 | 1 | 0.2 | Low | 0.0010 | >99.0000 |
| 0.15 | 1 | 0.25 | Low | 0.0010 | 3.0000 |
| 0.15 | 1 | 0.3 | Low | 0.0009 | >99.0000 |
| 0.15 | 1 | 0.35 | Low | 0.0011 | 6.0000 |
| 0.15 | 1 | 0.4 | Low | 0.0011 | >99.0000 |
| 0.15 | 30 | 0.05 | Low | 0.0038 | 0.6462 |
| 0.15 | 30 | 0.1 | Low | 0.0040 | 0.7034 |
| 0.15 | 30 | 0.15 | Low | 0.0039 | 0.6776 |
| 0.15 | 30 | 0.2 | Low | 0.0040 | 0.7253 |
| 0.15 | 30 | 0.25 | Low | 0.0040 | 0.7323 |
| 0.15 | 30 | 0.3 | Low | 0.0038 | 0.6802 |
| 0.15 | 30 | 0.35 | Low | 0.0040 | 0.7623 |
| 0.15 | 30 | 0.4 | Low | 0.0039 | 0.7410 |
| 0.15 | 200 | 0.05 | Low | 0.0098 | 0.1830 |
| 0.15 | 200 | 0.1 | Low | 0.0102 | 0.1901 |
| 0.15 | 200 | 0.15 | Low | 0.0096 | 0.1792 |
| 0.15 | 200 | 0.2 | Low | 0.0100 | 0.1883 |
| 0.15 | 200 | 0.25 | Low | 0.0097 | 0.1806 |
| 0.15 | 200 | 0.3 | Low | 0.0105 | 0.1937 |
| 0.15 | 200 | 0.35 | Low | 0.0102 | 0.1912 |
| 0.15 | 200 | 0.4 | Low | 0.0100 | 0.1871 |
| 0.15 | 430 | 0.05 | Low | 0.0156 | 0.1217 |
| 0.15 | 430 | 0.1 | Low | 0.0157 | 0.1224 |
| 0.15 | 430 | 0.15 | Low | 0.0162 | 0.1261 |
| 0.15 | 430 | 0.2 | Low | 0.0151 | 0.1180 |
| 0.15 | 430 | 0.25 | Low | 0.0155 | 0.1192 |
| 0.15 | 430 | 0.3 | Low | 0.0157 | 0.1217 |
| 0.15 | 430 | 0.35 | Low | 0.0162 | 0.1264 |
| 0.15 | 430 | 0.4 | Low | 0.0151 | 0.1169 |
| 0.25 | 1 | 0.05 | High | 0.0340 | >99.0000 |
| 0.25 | 1 | 0.1 | High | 0.0280 | >99.0000 |
| 0.25 | 1 | 0.15 | High | 0.0330 | >99.0000 |
| 0.25 | 1 | 0.2 | High | 0.0348 | >99.0000 |
| 0.25 | 1 | 0.25 | High | 0.0398 | >99.0000 |
| 0.25 | 1 | 0.3 | High | 0.0388 | >99.0000 |
| 0.25 | 1 | 0.35 | High | 0.0333 | >99.0000 |
| 0.25 | 1 | 0.4 | High | 0.0345 | >99.0000 |
| 0.25 | 30 | 0.05 | High | 0.0686 | 5.4390 |
| 0.25 | 30 | 0.1 | High | 0.0996 | 8.0848 |
| 0.25 | 30 | 0.15 | High | 0.1754 | 14.2467 |
| 0.25 | 30 | 0.2 | High | 0.2019 | 16.4404 |
| 0.25 | 30 | 0.25 | High | 0.2813 | 21.9251 |
| 0.25 | 30 | 0.3 | High | 0.3016 | 23.9697 |
| 0.25 | 30 | 0.35 | High | 0.3782 | 30.3054 |
| 0.25 | 30 | 0.4 | High | 0.4137 | 32.8201 |
| 0.25 | 200 | 0.05 | High | 0.3332 | 3.6400 |
| 0.25 | 200 | 0.1 | High | 0.6966 | 7.5607 |
| 0.25 | 200 | 0.15 | High | 1.0157 | 11.0998 |
| 0.25 | 200 | 0.2 | High | 1.3849 | 15.1449 |
| 0.25 | 200 | 0.25 | High | 1.7206 | 18.7576 |
| 0.25 | 200 | 0.3 | High | 2.0573 | 22.4518 |
| 0.25 | 200 | 0.35 | High | 2.4286 | 26.4826 |
| 0.25 | 200 | 0.4 | High | 2.7220 | 29.6788 |
| 0.25 | 430 | 0.05 | High | 0.8420 | 3.8935 |
| 0.25 | 430 | 0.1 | High | 1.6396 | 7.5727 |
| 0.25 | 430 | 0.15 | High | 2.4436 | 11.2980 |
| 0.25 | 430 | 0.2 | High | 3.2695 | 15.1148 |
| 0.25 | 430 | 0.25 | High | 4.0922 | 18.9098 |
| 0.25 | 430 | 0.3 | High | 4.8950 | 22.6163 |
| 0.25 | 430 | 0.35 | High | 5.6569 | 26.1324 |
| 0.25 | 430 | 0.4 | High | 6.3864 | 29.5008 |
| 0.25 | 1 | 0.05 | Medium | 0.0045 | >99.0000 |
| 0.25 | 1 | 0.1 | Medium | 0.0046 | >99.0000 |
| 0.25 | 1 | 0.15 | Medium | 0.0049 | >99.0000 |
| 0.25 | 1 | 0.2 | Medium | 0.0050 | >99.0000 |
| 0.25 | 1 | 0.25 | Medium | 0.0046 | >99.0000 |
| 0.25 | 1 | 0.3 | Medium | 0.0050 | >99.0000 |
| 0.25 | 1 | 0.35 | Medium | 0.0046 | >99.0000 |
| 0.25 | 1 | 0.4 | Medium | 0.0050 | >99.0000 |
| 0.25 | 30 | 0.05 | Medium | 0.0105 | 0.9364 |
| 0.25 | 30 | 0.1 | Medium | 0.0143 | 1.2493 |
| 0.25 | 30 | 0.15 | Medium | 0.0216 | 1.9087 |
| 0.25 | 30 | 0.2 | Medium | 0.0269 | 2.3521 |
| 0.25 | 30 | 0.25 | Medium | 0.0348 | 3.0042 |
| 0.25 | 30 | 0.3 | Medium | 0.0370 | 3.1199 |
| 0.25 | 30 | 0.35 | Medium | 0.0486 | 4.1280 |
| 0.25 | 30 | 0.4 | Medium | 0.0513 | 4.3419 |
| 0.25 | 200 | 0.05 | Medium | 0.0448 | 0.5075 |
| 0.25 | 200 | 0.1 | Medium | 0.0859 | 0.9611 |
| 0.25 | 200 | 0.15 | Medium | 0.1287 | 1.4310 |
| 0.25 | 200 | 0.2 | Medium | 0.1704 | 1.8876 |
| 0.25 | 200 | 0.25 | Medium | 0.2155 | 2.3841 |
| 0.25 | 200 | 0.3 | Medium | 0.2581 | 2.8572 |
| 0.25 | 200 | 0.35 | Medium | 0.3008 | 3.3182 |
| 0.25 | 200 | 0.4 | Medium | 0.3406 | 3.7584 |
| 0.25 | 430 | 0.05 | Medium | 0.1022 | 0.4814 |
| 0.25 | 430 | 0.1 | Medium | 0.2034 | 0.9524 |
| 0.25 | 430 | 0.15 | Medium | 0.3076 | 1.4380 |
| 0.25 | 430 | 0.2 | Medium | 0.4063 | 1.8951 |
| 0.25 | 430 | 0.25 | Medium | 0.5067 | 2.3614 |
| 0.25 | 430 | 0.3 | Medium | 0.6093 | 2.8398 |
| 0.25 | 430 | 0.35 | Medium | 0.7136 | 3.3264 |
| 0.25 | 430 | 0.4 | Medium | 0.8166 | 3.8055 |
| 0.25 | 1 | 0.05 | Low | 0.0011 | >99.0000 |
| 0.25 | 1 | 0.1 | Low | 0.0012 | >99.0000 |
| 0.25 | 1 | 0.15 | Low | 0.0012 | >99.0000 |
| 0.25 | 1 | 0.2 | Low | 0.0011 | >99.0000 |
| 0.25 | 1 | 0.25 | Low | 0.0011 | >99.0000 |
| 0.25 | 1 | 0.3 | Low | 0.0011 | >99.0000 |
| 0.25 | 1 | 0.35 | Low | 0.0011 | >99.0000 |
| 0.25 | 1 | 0.4 | Low | 0.0011 | >99.0000 |
| 0.25 | 30 | 0.05 | Low | 0.0048 | 0.4225 |
| 0.25 | 30 | 0.1 | Low | 0.0049 | 0.4246 |
| 0.25 | 30 | 0.15 | Low | 0.0049 | 0.4323 |
| 0.25 | 30 | 0.2 | Low | 0.0053 | 0.4447 |
| 0.25 | 30 | 0.25 | Low | 0.0054 | 0.4690 |
| 0.25 | 30 | 0.3 | Low | 0.0049 | 0.4202 |
| 0.25 | 30 | 0.35 | Low | 0.0050 | 0.4457 |
| 0.25 | 30 | 0.4 | Low | 0.0052 | 0.4597 |
| 0.25 | 200 | 0.05 | Low | 0.0128 | 0.1418 |
| 0.25 | 200 | 0.1 | Low | 0.0129 | 0.1433 |
| 0.25 | 200 | 0.15 | Low | 0.0127 | 0.1403 |
| 0.25 | 200 | 0.2 | Low | 0.0127 | 0.1413 |
| 0.25 | 200 | 0.25 | Low | 0.0129 | 0.1425 |
| 0.25 | 200 | 0.3 | Low | 0.0138 | 0.1530 |
| 0.25 | 200 | 0.35 | Low | 0.0130 | 0.1448 |
| 0.25 | 200 | 0.4 | Low | 0.0126 | 0.1400 |
| 0.25 | 430 | 0.05 | Low | 0.0196 | 0.0907 |
| 0.25 | 430 | 0.1 | Low | 0.0194 | 0.0900 |
| 0.25 | 430 | 0.15 | Low | 0.0215 | 0.0996 |
| 0.25 | 430 | 0.2 | Low | 0.0208 | 0.0963 |
| 0.25 | 430 | 0.25 | Low | 0.0202 | 0.0928 |
| 0.25 | 430 | 0.3 | Low | 0.0203 | 0.0936 |
| 0.25 | 430 | 0.35 | Low | 0.0211 | 0.0976 |
| 0.25 | 430 | 0.4 | Low | 0.0203 | 0.0934 |
| 0.35 | 1 | 0.05 | High | 0.0430 | >99.0000 |
| 0.35 | 1 | 0.1 | High | 0.0501 | >99.0000 |
| 0.35 | 1 | 0.15 | High | 0.0512 | >99.0000 |
| 0.35 | 1 | 0.2 | High | 0.0511 | >99.0000 |
| 0.35 | 1 | 0.25 | High | 0.0531 | >99.0000 |
| 0.35 | 1 | 0.3 | High | 0.0536 | >99.0000 |
| 0.35 | 1 | 0.35 | High | 0.0433 | >99.0000 |
| 0.35 | 1 | 0.4 | High | 0.0502 | >99.0000 |
| 0.35 | 30 | 0.05 | High | 0.0965 | 5.2976 |
| 0.35 | 30 | 0.1 | High | 0.1455 | 7.9686 |
| 0.35 | 30 | 0.15 | High | 0.2504 | 13.8517 |
| 0.35 | 30 | 0.2 | High | 0.2882 | 15.6922 |
| 0.35 | 30 | 0.25 | High | 0.3885 | 21.3492 |
| 0.35 | 30 | 0.3 | High | 0.4332 | 23.7575 |
| 0.35 | 30 | 0.35 | High | 0.5432 | 29.9861 |
| 0.35 | 30 | 0.4 | High | 0.5715 | 31.5488 |
| 0.35 | 200 | 0.05 | High | 0.4591 | 3.5421 |
| 0.35 | 200 | 0.1 | High | 0.9695 | 7.4848 |
| 0.35 | 200 | 0.15 | High | 1.4167 | 10.9260 |
| 0.35 | 200 | 0.2 | High | 1.9120 | 14.7429 |
| 0.35 | 200 | 0.25 | High | 2.3843 | 18.3864 |
| 0.35 | 200 | 0.3 | High | 2.8903 | 22.3049 |
| 0.35 | 200 | 0.35 | High | 3.3956 | 26.2188 |
| 0.35 | 200 | 0.4 | High | 3.8298 | 29.5491 |
| 0.35 | 430 | 0.05 | High | 1.1627 | 3.8287 |
| 0.35 | 430 | 0.1 | High | 2.2660 | 7.4535 |
| 0.35 | 430 | 0.15 | High | 3.4528 | 11.3702 |
| 0.35 | 430 | 0.2 | High | 4.5471 | 14.9676 |
| 0.35 | 430 | 0.25 | High | 5.6676 | 18.6440 |
| 0.35 | 430 | 0.3 | High | 6.8484 | 22.5355 |
| 0.35 | 430 | 0.35 | High | 7.9604 | 26.2063 |
| 0.35 | 430 | 0.4 | High | 9.0534 | 29.7958 |
| 0.35 | 1 | 0.05 | Medium | 0.0060 | >99.0000 |
| 0.35 | 1 | 0.1 | Medium | 0.0074 | >99.0000 |
| 0.35 | 1 | 0.15 | Medium | 0.0060 | >99.0000 |
| 0.35 | 1 | 0.2 | Medium | 0.0063 | >99.0000 |
| 0.35 | 1 | 0.25 | Medium | 0.0063 | >99.0000 |
| 0.35 | 1 | 0.3 | Medium | 0.0064 | >99.0000 |
| 0.35 | 1 | 0.35 | Medium | 0.0065 | >99.0000 |
| 0.35 | 1 | 0.4 | Medium | 0.0065 | >99.0000 |
| 0.35 | 30 | 0.05 | Medium | 0.0136 | 0.7874 |
| 0.35 | 30 | 0.1 | Medium | 0.0191 | 1.1011 |
| 0.35 | 30 | 0.15 | Medium | 0.0303 | 1.7267 |
| 0.35 | 30 | 0.2 | Medium | 0.0376 | 2.1447 |
| 0.35 | 30 | 0.25 | Medium | 0.0430 | 2.6413 |
| 0.35 | 30 | 0.3 | Medium | 0.0526 | 2.9537 |
| 0.35 | 30 | 0.35 | Medium | 0.0677 | 3.7853 |
| 0.35 | 30 | 0.4 | Medium | 0.0708 | 3.9546 |
| 0.35 | 200 | 0.05 | Medium | 0.0584 | 0.4627 |
| 0.35 | 200 | 0.1 | Medium | 0.1197 | 0.9389 |
| 0.35 | 200 | 0.15 | Medium | 0.1775 | 1.3867 |
| 0.35 | 200 | 0.2 | Medium | 0.2401 | 1.8722 |
| 0.35 | 200 | 0.25 | Medium | 0.2982 | 2.3236 |
| 0.35 | 200 | 0.3 | Medium | 0.3606 | 2.8043 |
| 0.35 | 200 | 0.35 | Medium | 0.4202 | 3.2671 |
| 0.35 | 200 | 0.4 | Medium | 0.4844 | 3.7662 |
| 0.35 | 430 | 0.05 | Medium | 0.1447 | 0.4815 |
| 0.35 | 430 | 0.1 | Medium | 0.2852 | 0.9454 |
| 0.35 | 430 | 0.15 | Medium | 0.4311 | 1.4276 |
| 0.35 | 430 | 0.2 | Medium | 0.5640 | 1.8652 |
| 0.35 | 430 | 0.25 | Medium | 0.7119 | 2.3524 |
| 0.35 | 430 | 0.3 | Medium | 0.8432 | 2.7859 |
| 0.35 | 430 | 0.35 | Medium | 0.9992 | 3.3013 |
| 0.35 | 430 | 0.4 | Medium | 1.1353 | 3.7478 |
| 0.35 | 1 | 0.05 | Low | 0.0012 | >99.0000 |
| 0.35 | 1 | 0.1 | Low | 0.0012 | >99.0000 |
| 0.35 | 1 | 0.15 | Low | 0.0012 | >99.0000 |
| 0.35 | 1 | 0.2 | Low | 0.0012 | >99.0000 |
| 0.35 | 1 | 0.25 | Low | 0.0011 | >99.0000 |
| 0.35 | 1 | 0.3 | Low | 0.0011 | >99.0000 |
| 0.35 | 1 | 0.35 | Low | 0.0012 | >99.0000 |
| 0.35 | 1 | 0.4 | Low | 0.0012 | >99.0000 |
| 0.35 | 30 | 0.05 | Low | 0.0058 | 0.3235 |
| 0.35 | 30 | 0.1 | Low | 0.0059 | 0.3190 |
| 0.35 | 30 | 0.15 | Low | 0.0060 | 0.3306 |
| 0.35 | 30 | 0.2 | Low | 0.0057 | 0.3110 |
| 0.35 | 30 | 0.25 | Low | 0.0055 | 0.2980 |
| 0.35 | 30 | 0.3 | Low | 0.0057 | 0.3177 |
| 0.35 | 30 | 0.35 | Low | 0.0058 | 0.3195 |
| 0.35 | 30 | 0.4 | Low | 0.0058 | 0.3168 |
| 0.35 | 200 | 0.05 | Low | 0.0154 | 0.1207 |
| 0.35 | 200 | 0.1 | Low | 0.0161 | 0.1269 |
| 0.35 | 200 | 0.15 | Low | 0.0158 | 0.1237 |
| 0.35 | 200 | 0.2 | Low | 0.0156 | 0.1226 |
| 0.35 | 200 | 0.25 | Low | 0.0160 | 0.1260 |
| 0.35 | 200 | 0.3 | Low | 0.0156 | 0.1220 |
| 0.35 | 200 | 0.35 | Low | 0.0158 | 0.1238 |
| 0.35 | 200 | 0.4 | Low | 0.0149 | 0.1167 |
| 0.35 | 430 | 0.05 | Low | 0.0231 | 0.0757 |
| 0.35 | 430 | 0.1 | Low | 0.0244 | 0.0796 |
| 0.35 | 430 | 0.15 | Low | 0.0238 | 0.0782 |
| 0.35 | 430 | 0.2 | Low | 0.0230 | 0.0753 |
| 0.35 | 430 | 0.25 | Low | 0.0230 | 0.0756 |
| 0.35 | 430 | 0.3 | Low | 0.0232 | 0.0762 |
| 0.35 | 430 | 0.35 | Low | 0.0237 | 0.0779 |
| 0.35 | 430 | 0.4 | Low | 0.0243 | 0.0797 |
| 0.45 | 1 | 0.05 | High | 0.0568 | >99.0000 |
| 0.45 | 1 | 0.1 | High | 0.0668 | >99.0000 |
| 0.45 | 1 | 0.15 | High | 0.0616 | >99.0000 |
| 0.45 | 1 | 0.2 | High | 0.0645 | >99.0000 |
| 0.45 | 1 | 0.25 | High | 0.0692 | >99.0000 |
| 0.45 | 1 | 0.3 | High | 0.0635 | >99.0000 |
| 0.45 | 1 | 0.35 | High | 0.0556 | >99.0000 |
| 0.45 | 1 | 0.4 | High | 0.0659 | >99.0000 |
| 0.45 | 30 | 0.05 | High | 0.1210 | 5.0927 |
| 0.45 | 30 | 0.1 | High | 0.1825 | 7.6740 |
| 0.45 | 30 | 0.15 | High | 0.3111 | 13.2555 |
| 0.45 | 30 | 0.2 | High | 0.3635 | 15.1544 |
| 0.45 | 30 | 0.25 | High | 0.5013 | 21.0582 |
| 0.45 | 30 | 0.3 | High | 0.5625 | 23.4805 |
| 0.45 | 30 | 0.35 | High | 0.6908 | 29.0631 |
| 0.45 | 30 | 0.4 | High | 0.7269 | 30.5439 |
| 0.45 | 200 | 0.05 | High | 0.5976 | 3.6057 |
| 0.45 | 200 | 0.1 | High | 1.2575 | 7.5802 |
| 0.45 | 200 | 0.15 | High | 1.8335 | 11.0346 |
| 0.45 | 200 | 0.2 | High | 2.4875 | 14.9924 |
| 0.45 | 200 | 0.25 | High | 3.0689 | 18.4612 |
| 0.45 | 200 | 0.3 | High | 3.6875 | 22.1928 |
| 0.45 | 200 | 0.35 | High | 4.3519 | 26.1992 |
| 0.45 | 200 | 0.4 | High | 4.9484 | 29.7833 |
| 0.45 | 430 | 0.05 | High | 1.4780 | 3.7839 |
| 0.45 | 430 | 0.1 | High | 2.9161 | 7.4616 |
| 0.45 | 430 | 0.15 | High | 4.4022 | 11.2585 |
| 0.45 | 430 | 0.2 | High | 5.8528 | 14.9747 |
| 0.45 | 430 | 0.25 | High | 7.3103 | 18.6967 |
| 0.45 | 430 | 0.3 | High | 8.8173 | 22.5468 |
| 0.45 | 430 | 0.35 | High | 10.2188 | 26.1388 |
| 0.45 | 430 | 0.4 | High | 11.6463 | 29.7931 |
| 0.45 | 1 | 0.05 | Medium | 0.0082 | >99.0000 |
| 0.45 | 1 | 0.1 | Medium | 0.0089 | >99.0000 |
| 0.45 | 1 | 0.15 | Medium | 0.0080 | >99.0000 |
| 0.45 | 1 | 0.2 | Medium | 0.0084 | >99.0000 |
| 0.45 | 1 | 0.25 | Medium | 0.0080 | >99.0000 |
| 0.45 | 1 | 0.3 | Medium | 0.0078 | >99.0000 |
| 0.45 | 1 | 0.35 | Medium | 0.0082 | >99.0000 |
| 0.45 | 1 | 0.4 | Medium | 0.0082 | >99.0000 |
| 0.45 | 30 | 0.05 | Medium | 0.0168 | 0.7671 |
| 0.45 | 30 | 0.1 | Medium | 0.0237 | 1.0637 |
| 0.45 | 30 | 0.15 | Medium | 0.0386 | 1.7138 |
| 0.45 | 30 | 0.2 | Medium | 0.0476 | 2.0943 |
| 0.45 | 30 | 0.25 | Medium | 0.0632 | 2.7588 |
| 0.45 | 30 | 0.3 | Medium | 0.0682 | 2.9684 |
| 0.45 | 30 | 0.35 | Medium | 0.0868 | 3.7641 |
| 0.45 | 30 | 0.4 | Medium | 0.0921 | 4.0109 |
| 0.45 | 200 | 0.05 | Medium | 0.0748 | 0.4575 |
| 0.45 | 200 | 0.1 | Medium | 0.1550 | 0.9406 |
| 0.45 | 200 | 0.15 | Medium | 0.2302 | 1.3914 |
| 0.45 | 200 | 0.2 | Medium | 0.3108 | 1.8783 |
| 0.45 | 200 | 0.25 | Medium | 0.3848 | 2.3212 |
| 0.45 | 200 | 0.3 | Medium | 0.4617 | 2.7826 |
| 0.45 | 200 | 0.35 | Medium | 0.5399 | 3.2549 |
| 0.45 | 200 | 0.4 | Medium | 0.6236 | 3.7566 |
| 0.45 | 430 | 0.05 | Medium | 0.1859 | 0.4797 |
| 0.45 | 430 | 0.1 | Medium | 0.3657 | 0.9404 |
| 0.45 | 430 | 0.15 | Medium | 0.5524 | 1.4194 |
| 0.45 | 430 | 0.2 | Medium | 0.7286 | 1.8702 |
| 0.45 | 430 | 0.25 | Medium | 0.9158 | 2.3499 |
| 0.45 | 430 | 0.3 | Medium | 1.0876 | 2.7901 |
| 0.45 | 430 | 0.35 | Medium | 1.2852 | 3.2967 |
| 0.45 | 430 | 0.4 | Medium | 1.4648 | 3.7555 |
| 0.45 | 1 | 0.05 | Low | 0.0012 | >99.0000 |
| 0.45 | 1 | 0.1 | Low | 0.0012 | >99.0000 |
| 0.45 | 1 | 0.15 | Low | 0.0013 | >99.0000 |
| 0.45 | 1 | 0.2 | Low | 0.0012 | >99.0000 |
| 0.45 | 1 | 0.25 | Low | 0.0012 | >99.0000 |
| 0.45 | 1 | 0.3 | Low | 0.0012 | >99.0000 |
| 0.45 | 1 | 0.35 | Low | 0.0013 | >99.0000 |
| 0.45 | 1 | 0.4 | Low | 0.0012 | >99.0000 |
| 0.45 | 30 | 0.05 | Low | 0.0063 | 0.2676 |
| 0.45 | 30 | 0.1 | Low | 0.0065 | 0.2702 |
| 0.45 | 30 | 0.15 | Low | 0.0065 | 0.2692 |
| 0.45 | 30 | 0.2 | Low | 0.0064 | 0.2663 |
| 0.45 | 30 | 0.25 | Low | 0.0065 | 0.2701 |
| 0.45 | 30 | 0.3 | Low | 0.0062 | 0.2636 |
| 0.45 | 30 | 0.35 | Low | 0.0065 | 0.2733 |
| 0.45 | 30 | 0.4 | Low | 0.0061 | 0.2550 |
| 0.45 | 200 | 0.05 | Low | 0.0174 | 0.1061 |
| 0.45 | 200 | 0.1 | Low | 0.0177 | 0.1082 |
| 0.45 | 200 | 0.15 | Low | 0.0177 | 0.1078 |
| 0.45 | 200 | 0.2 | Low | 0.0176 | 0.1072 |
| 0.45 | 200 | 0.25 | Low | 0.0174 | 0.1057 |
| 0.45 | 200 | 0.3 | Low | 0.0173 | 0.1051 |
| 0.45 | 200 | 0.35 | Low | 0.0168 | 0.1024 |
| 0.45 | 200 | 0.4 | Low | 0.0169 | 0.1026 |
| 0.45 | 430 | 0.05 | Low | 0.0258 | 0.0657 |
| 0.45 | 430 | 0.1 | Low | 0.0268 | 0.0682 |
| 0.45 | 430 | 0.15 | Low | 0.0276 | 0.0706 |
| 0.45 | 430 | 0.2 | Low | 0.0256 | 0.0654 |
| 0.45 | 430 | 0.25 | Low | 0.0256 | 0.0653 |
| 0.45 | 430 | 0.3 | Low | 0.0256 | 0.0654 |
| 0.45 | 430 | 0.35 | Low | 0.0263 | 0.0673 |
| 0.45 | 430 | 0.4 | Low | 0.0260 | 0.0665 |

Table S3. Summary of population-differentiated genes for each tissue.

| Gene expression models | Number of significant genes | Number of genes | Ratio |
| --- | --- | --- | --- |
| Adipose_Subcutaneous | 7433 | 8650 | 0.8593 |
| Adipose_Visceral_Omentum | 6389 | 7340 | 0.8704 |
| Adrenal_Gland | 4247 | 4843 | 0.8769 |
| Artery_Aorta | 6583 | 7599 | 0.8663 |
| Artery_Coronary | 3515 | 4046 | 0.8688 |
| Artery_Tibial | 7410 | 8615 | 0.8601 |
| Brain_Amygdala | 2438 | 2787 | 0.8748 |
| Brain_Anterior_cingulate_cortex_BA24 | 3086 | 3544 | 0.8708 |
| Brain_Caudate_basal_ganglia | 4376 | 5004 | 0.8745 |
| Brain_Cerebellar_Hemisphere | 5030 | 5753 | 0.8743 |
| Brain_Cerebellum | 5903 | 6794 | 0.8689 |
| Brain_Cortex | 4808 | 5500 | 0.8741 |
| Brain_Frontal_Cortex_BA9 | 4005 | 4563 | 0.8777 |
| Brain_Hippocampus | 3208 | 3688 | 0.8698 |
| Brain_Hypothalamus | 3224 | 3652 | 0.8828 |
| Brain_Nucleus_accumbens_basal_ganglia | 4238 | 4851 | 0.8736 |
| Brain_Putamen_basal_ganglia | 3902 | 4436 | 0.8796 |
| Brain_Spinal_cord_cervical_c-1 | 2827 | 3250 | 0.8698 |
| Brain_Substantia_nigra | 2242 | 2559 | 0.8761 |
| Breast_Mammary_Tissue | 5652 | 6461 | 0.8748 |
| Cells_Cultured_fibroblasts | 7735 | 8933 | 0.8659 |
| Cells_EBV-transformed_lymphocytes | 2554 | 2904 | 0.8795 |
| Colon_Sigmoid | 5375 | 6173 | 0.8707 |
| Colon_Transverse | 5535 | 6304 | 0.8780 |
| Esophagus_Gastroesophageal_Junction | 5467 | 6291 | 0.8690 |
| Esophagus_Mucosa | 7390 | 8521 | 0.8673 |
| Esophagus_Muscularis | 7128 | 8231 | 0.8660 |
| Heart_Atrial_Appendage | 5759 | 6641 | 0.8672 |
| Heart_Left_Ventricle | 5265 | 6013 | 0.8756 |
| Kidney_Cortex | 1441 | 1642 | 0.8776 |
| Liver | 3320 | 3773 | 0.8799 |
| Lung | 6898 | 7969 | 0.8656 |
| Minor_Salivary_Gland | 2560 | 2916 | 0.8779 |
| Muscle_Skeletal | 6544 | 7583 | 0.8630 |
| Nerve_Tibial | 8640 | 10012 | 0.8630 |
| Ovary | 3125 | 3587 | 0.8712 |
| Pancreas | 5120 | 5896 | 0.8684 |
| Pituitary | 4969 | 5688 | 0.8736 |
| Prostate | 3719 | 4302 | 0.8645 |
| Skin_Not_Sun_Exposed_Suprapubic | 7427 | 8650 | 0.8586 |
| Skin_Sun_Exposed_Lower_leg | 8008 | 9299 | 0.8612 |
| Small_Intestine_Terminal_Ileum | 3235 | 3670 | 0.8815 |
| Spleen | 5032 | 5774 | 0.8715 |
| Stomach | 4507 | 5156 | 0.8741 |
| Testis | 8562 | 9978 | 0.8581 |
| Thyroid | 8284 | 9652 | 0.8583 |
| Uterus | 2235 | 2541 | 0.8796 |
| Vagina | 2256 | 2562 | 0.8806 |
| Whole_Blood | 6317 | 7252 | 0.8711 |

Table S4. The correlation summary between predicted Gene expression using SNP data via PrediXcan method (GE_PrediXcan_) and normalized real gene expression GE_Real_(normalized) values from non-smoking lung cancer patients in Taiwan

| **Correlation summary** | |
| --- | --- |
| Minimum | -0.780 |
| 1^st^ Quartile | -0.178 |
| Median | -0.052 |
| Mean | -0.058 |
| 3^rd^ Quartile | 0.065 |
| Max | 0.700 |
