# Supplementary material for: Cross-population enhancement of PrediXcan predictions with a gnomAD-based east Asian reference framework
Source: Brief Bioinform. 2024 Oct 23;25(6):bbae549. doi: 10.1093/bib/bbae549 (PMC11497844; doi:10.1093/bib/bbae549)
Supplement: PreditAP_turorial0831_bbae549 [file preditap_turorial0831_bbae549.pdf]

PredictAP Tutorial

1. Prerequisites

- Install the PredictAP package

  To run PredictAP, you will need R version 4.3.2 or higher and the PredictAP package installed locally. You can download the PredictAP package file from the following link: [PredictAP Download].

- The gene expression prediction file from PrediXcan

  After running the PrediXcan algorithm (using the GTEx v8 Elastic net model), you will obtain a standard PrediXcan prediction file. For example, we applied PrediXcan to predict gene expression in lung tissue using GSE33356 lung data. The generated file is shown below:

	FID	IID	ENSG00000000460.16	ENSG00000000938.12	ENSG00000000971.15	ENSG00000001167.14	ENSG00000001460.17
1	GSM824988	GSM824988	-0.1398644	0	0.05829280	0.3780235	-0.06092589
2	GSM824990	GSM824990	-0.1398644	0	0.13453709	0.3112629	-0.05063678
3	GSM824992	GSM824992	-0.1398644	0	0.13453709	0.2099854	-0.04868573
4	GSM824994	GSM824994	-0.1398644	0	-0.01795150	0.2899145	-0.04731808
5	GSM824996	GSM824996	-0.1188632	0	-0.01795150	0.2490042	-0.04061628
6	GSM824998	GSM824998	-0.1398644	0	0.04075691	0.3461594	-0.10973238
7	GSM825000	GSM825000	-0.1410336	0	0.05829280	0.2617045	-0.02828219
8	GSM825002	GSM825002	-0.1398644	0	0.04075691	0.1848165	-0.13051717
9	GSM825004	GSM825004	-0.1410336	0	0.13453709	0.1932927	-0.03719066
10	GSM825006	GSM825006	-0.1386952	0	0.13453709	0.2193389	-0.02948815

2. Example

Here we take GSE33356 as an example, to demonstrate how to use the package function:

```r
#Set up
library(PredictAP)
library(data.table)
#Read data
mydata <- fread("GSE33356_predicted_expression.txt")
#Run
output <- rankcal(data = mydata, tissue = "x32")
```

The *rankcal* function has two arguments: the first one is your dataset name, and the second one is the tissue type you predicted with PrediXcan. In this case, we predicted GSE33356 gene expression in lung tissue using PrediXcan, so we choose "x32" (lung).

Below is the full list of possible values:

Value	Tissue	Value	Tissue
“x1”	Adipose_Subcutaneous	“x26”	Esophagus_Mucosa
“x2”	Adipose_Visceral_Omentum	“x27”	Esophagus_Muscularis
“x3”	Adrenal_Gland	“x28”	Heart_Atrial_Appendage
“x4”	Artery_Aorta	“x29”	Heart_Left_Ventricle
“x5”	Artery_Coronary	“x30”	Kidney_Cortex
“x6”	Artery_Tibial	“x31”	Liver
“x7”	Brain_Amygdala	“x32”	Lung
“x8”	Brain_Anterior_cingulate_cortex_BA24	“x33”	Minor_Salivary_Gland
“x9”	Brain_Caudate_basal_ganglia	“x34”	Muscle_Skeletal
“x10”	Brain_Cerebellar_Hemisphere	“x35”	Nerve_Tibial
“x11”	Brain_Cerebellum	“x36”	Ovary
“x12”	Brain_Cortex	“x37”	Pancreas
“x13”	Brain_Frontal_Cortex_BA9	“x38”	Pituitary
“x14”	Brain_Hippocampus	“x39”	Prostate
“x15”	Brain_Hypothalamus	“x40”	Skin_Not_Sun_Exposed_Suprapubic
“x16”	Brain_Nucleus_accumbens_basal_ganglia	“x41”	Skin_Sun_Exposed_Lower_leg
“x17”	Brain_Putamen_basal_ganglia	“x42”	Small_Intestine_Terminal_Ileum
“x18”	Brain_Spinal_cord_cervical_c-1	“x43”	Spleen
“x19”	Brain_Substantia_nigra	“x44”	Stomach
“x20”	Breast_Mammary_Tissue	“x45”	Testis
“x21”	Cells_Cultured_fibroblasts	“x46”	Thyroid
“x22”	Cells_EBV-transformed_lymphocytes	“x47”	Uterus
“x23”	Colon_Sigmoid	“x48”	Vagina
“x24”	Colon_Transverse	“x49”	Whole_Blood
“x25”	Esophagus_Gastroesophageal_Junction		

The final result is shown below:

FID	IID	gene	expression	percentile.rank	N.snps	mean	sd	ks.test	NFE.EAS.mean.diff
GSM824988	GSM824988	ENSG00000000460.16	−0.139864390	0.0	10	−2.631175e−03	0.007610846	Sig.	−97.2%
GSM824988	GSM824988	ENSG00000000938.12	0.000000000	95.0	6	−5.380984e−05	0.001811622	Sig.	−99.1%
GSM824988	GSM824988	ENSG00000000971.15	0.058292795	91.2	6	3.500955e−03	0.036966013	Nonsig.	−72.8%
GSM824988	GSM824988	ENSG00000001167.14	0.378023546	100.0	111	−2.050286e−02	0.055607412	Sig.	−257.2%
GSM824988	GSM824988	ENSG00000001460.17	−0.060925887	100.0	16	−1.633135e−01	0.028396812	Sig.	56.6%
GSM824988	GSM824988	ENSG00000001561.6	0.170480658	59.8	41	1.607835e−01	0.040465681	Sig.	−28%
GSM824988	GSM824988	ENSG00000001617.11	−0.004725254	100.0	29	−1.050122e−01	0.031572787	Sig.	152%
GSM824988	GSM824988	ENSG00000002016.17	−0.082619909	7.4	17	2.501919e−03	0.053917751	Sig.	−108.1%
GSM824988	GSM824988	ENSG00000002549.12	0.023949230	100.0	17	−2.279398e−01	0.022102354	Sig.	42.4%
GSM824988	GSM824988	ENSG00000002726.20	−0.139730536	69.0	5	−1.613890e−01	0.041947401	Nonsig.	−1.4%

The first four columns represent the original data, and columns 5–10 demonstrate the results for users to evaluate the prediction of gene expression by PredictAP.

Variable	Description
FID	Family ID from the user's data
IID	Individual ID from the user's data
gene	Ensembl gene ID
expression	Predicted gene expression from the user's data
percentile.rank	Percentile ranks of user's gene expression compared to the reference
N.snps	Number of variants used in predicting gene expression
mean	Average value of the reference
sd	Standard deviation of the reference
Ks.test	Significance indicator of the Kolmogorov-Smirnov test to determine if the gene has different expression between EAS and NFE populations
NFE.EAS.mean.diff	Mean differences between EAS and NFE reference populations
